# Modulation of Inducible Nitric Oxide Synthase Expression in LPS-Stimulated BV-2 Microglia by Prenylated Chalcones from *Cullen corylifolium* (L.) Medik. through Inhibition of I-κBα Degradation

**DOI:** 10.3390/molecules23010109

**Published:** 2018-01-04

**Authors:** Do Hee Kim, Hua Li, Yeong Eun Han, Ji Hye Jeong, Hwa Jin Lee, Jae-Ha Ryu

**Affiliations:** 1 Research Institute of Pharmaceutical Sciences, College of Pharmacy, Sookmyung Women’s University, Seoul 04310, Korea; kdhkazan3@naver.com (D.H.K.); cooldog227@hotmail.com (H.L.); thddl1428@naver.com (Y.E.H.); jjh4415@naver.com (J.H.J.); 2 Department of Natural Medicine Resources, Semyung University, Jecheon 27136, Korea

**Keywords:** *Cullen corylifolium*, *Psoralea corylifolia*, prenylated chalcone, nitric oxide, inducible nitric oxide synthase, prostaglandin E2, inhibitory-κBα

## Abstract

The overproduction of nitric oxide (NO) and prostaglandin E_2_ (PGE_2_) by microglia may cause neurodegenerative diseases, such as Alzheimer’s disease and Parkinson’s disease. From the activity-guided purification of *Cullen corylifolium* (L.) Medik. (syn. *Psoralea corylifolia* L.), three prenylated chalcones were identified: isobavachalcone (**1**), bavachromene (**2**), and kanzonol B (**3**). These prenylated chalcones showed concentration-dependent inhibitory effects on NO and PGE_2_ production in lipopolysaccharide (LPS)-activated microglia. Western blotting and RT-PCR analysis demonstrated that these prenylchalcones reduced the expression of protein and mRNA of inducible nitric oxide synthase (iNOS) and cyclooxygenase-2 (COX-2) in LPS-activated microglia. Furthermore, three prenylated chalcones blocked the inhibitory-κBα (I-κBα) degradation and down-regulated nuclear factor κB (NF-κB) level of nucleus in LPS-stimulated BV-2 microglia. Therefore, these prenylated chalcones from *Psoralea corylifolia* may be beneficial for the treatment of neuro-inflammatory diseases by modulating iNOS and COX-2 expressions in activated microglial cells.

## 1. Introduction

Microglia, an innate immune cell and resident phagocyte in the central nervous system (CNS), plays an active role in CNS homeostasis, infection and pathogen defense. Activated microglia produce pro-inflammatory and neuro-toxic materials such as nitric oxide (NO), prostaglandins (PGs) and superoxide anion [1]. These mediators are involved in the brain injuries and neurodegenerative diseases including Alzheimer’s disease, amyotrophic lateral sclerosis and Parkinson’s disease [2,3]. NO can be produced by three isoforms of nitric oxide synthase (NOS) such as neuronal NOS (nNOS), endothelial NOS (eNOS) and inducible NOS (iNOS) [4]. The nNOS and eNOS, constitutive NOS (cNOS), release small amounts of NO and maintain physiological functions, while iNOS, inducible form, by lipopolysaccharide (LPS) and various cytokines, produces micromolar levels of NO [5]. Many reports have shown that the iNOS and a large amount of NO produced by activated microglia contribute to the progress of neurodegenerative diseases [6]. PGE_2_, the other inflammatory mediator, is synthesized by a cyclooxygenase (COX)-dependent pathway [7]. COX enzyme consists of at least two isoforms, namely cyclooxygenase-1 (COX-1) and cyclooxygenase-2 (COX-2). The COX-2 form is induced by pro-inflammatory effectors such as cytokines and LPS and leads to an elevated production of PGE_2_, whereas COX-1 is constitutively expressed under normal conditions [8]. Therefore, inhibition of pro-inflammatory enzymes may have therapeutic effects for the treatment of diverse diseases [9,10]. The seeds of *Cullen corylifolium* (L.) Medik. (syn. *Psoralea corylifolia* L.) (Leguminosae) have been used traditionally for treatment of gynaecological bleeding, vitiligo and psoriasis. Its seeds were reported to contain coumarins, flavonoids and meroterpene phenols, and some of them exhibited anti-neuroinflammatory, anti-bacterial, anti-tumor, broadening coronary artery and estrogen-like activities [11,12,13,14,15,16,17]. The present study reports that prenylated chalcones from the extracts of *C. corylifolium* display inhibitory activities on NO and PGE_2_ production in LPS-activated microglia, and discloses the underlying mechanism for their biological activities.

## 2. Results and Discussion

The prenylated chalcones **1**–**3** were isolated from *Cullen corylifolium* by activity-guided purification. Their chemical structures were elucidated by the analysis of their mass and NMR spectral data (Supplementary Materials, Figures S1–S15). Isobavachalcone (**1**) [18,19], has been predominantly isolated from plants of *Leguminosae* and *Moraceae* families [20] and has a prenyl moiety, while bavachromene (**2**) [12,21] and kanzonol B (**3**) [22], have cyclized prenyl moieties that form a pyrano ring in the chalcone skeleton (Figure 1).

**Figure 2:**
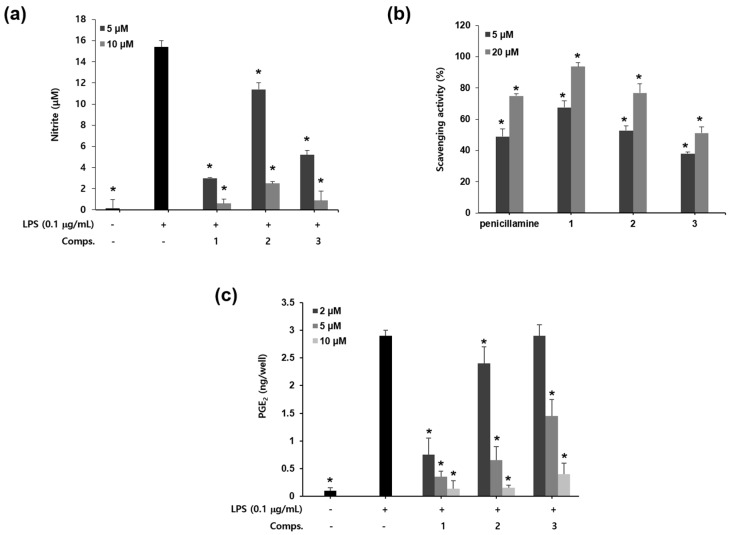
The effects of compounds **1**–**3** on pro-inflammatory and neuro-toxic mediators. (**a**) Inhibitory effects of compounds **1**–**3** on NO production in LPS-stimulated BV-2 microglia. The amount of NO in culture medium was measured using Griess reagents; (**b**) Peroxynitrite (ONOO^−^) scavenging activity of compounds **1**–**3** in vitro cell free system. The level of ONOO^−^ was measured by detecting the oxidation of DHR123 as experimental section. Penicillamine was used as a positive control; (**c**) Inhibitory effects of compounds **1**–**3** on PGE_2_ production in LPS-stimulated BV-2 microglia. The levels of PGE_2_ were determined by the enzyme immunoassay. The values are expressed as the means ± S.D. of three experiments. * *p* < 0.05.

Isolated compounds showed concentration-dependent inhibitory activity on NO production in LPS-activated microglia (Figure 2a). Their IC_50_ values were 1.6 ± 0.11 μM (**1**), 2.4 ± 0.18 μM (**2**), and 2.2 ± 0.21 μM (**3**), respectively. Compounds **1**–**3** did not affect cell viability at the tested concentrations (data not shown). In particular, the chalcone **1** characterized by the presence of open chain prenyl group showed more potent inhibitory activity on NO production than chalcones **2** and **3** containing cyclic pyranoprenyl groups.

NO combines with superoxide anion and is transformed into peroxynitrite (ONOO^−^), an inflammatory mediator with strong oxidizing properties [23]. Peroxynitrite is responsible for the pathogenesis of neuronal diseases such as neuronal ageing and Alzheimer’s disease [24]. To examine whether prenylated chalcones **1**–**3** could remove peroxynitrite, its scavenging was measured in a cell free assay system by observing the fluorescence of the oxidized substance of dihydrorhodamine 123 (DHR 123) by authentic peroxynitrite [25]. Compounds **1**–**3** showed potent ONOO^−^ scavenging activity (93.6%, 76.8% and 51.2% at 20 μM, Figure 2b), respectively, while penicillamine (20 μM), a positive control, displayed 74.9% scavenging activity. Prenylated chalcone **1** showed more efficient scavenging activity compared with cyclic pyranoprenyl group-containing chalcones **2** and **3**. Taken together, these results show that active compounds **1**–**3** downregulate the overproduction of NO in activated microglial cells and also significantly eliminate the ONOO^−^, which suggest that compounds **1**–**3** might prevent the neuro-inflammatory response.

Prostaglandin E_2_ (PGE_2_) is a critical inflammatory mediator, which is a major material produced by COX-2 [7]. To determine whether compounds **1**–**3** inhibit PGE_2_ production in LPS-induced BV-2 microglia, we examined the PGE_2_ accumulation using PGE_2_ enzyme immunoassay (EIA). Compounds **1**–**3** (5 μM) showed 90%, 78% and 54% inhibition of PGE_2_ production, respectively (Figure 2c). The results showed that LPS enhanced the production of PGE_2_ in BV-2 cells and prenylated chalcones **1**–**3** significantly suppressed the PGE_2_ synthesis. Furthermore, prenyl chalcone **1** treatment showed lower levels of PGE_2_ synthesis than **2** and **3** with cyclic pyranoprenyl chalcone groups.

**Figure 3:**
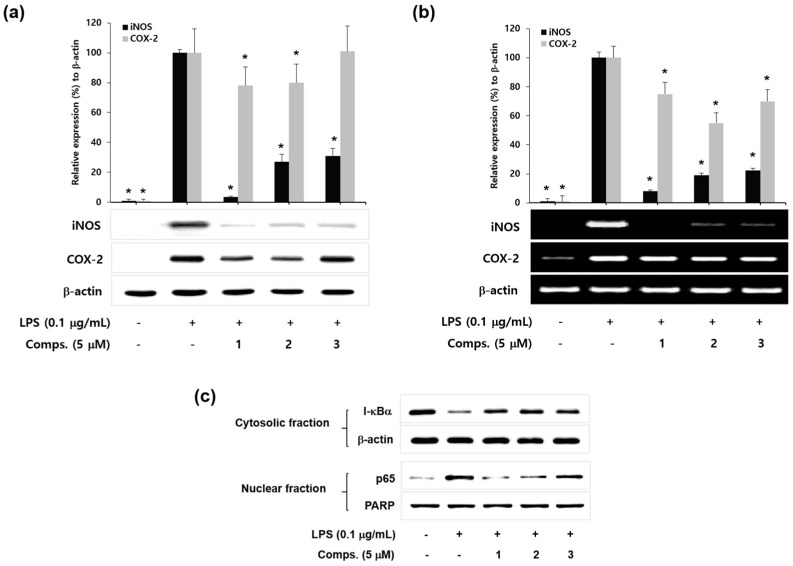
The effects of compounds **1**–**3** on LPS-induced iNOS/COX-2 expression and I-κBα degradation in BV-2 microglia. (**a**) The effects of compounds **1**–**3** on LPS-stimulated iNOS and COX-2 protein expression. The protein levels were determined by Western blot analysis. The relative intensity of iNOS/COX-2 to β-actin bands was measured by densitometry. The values represent the means ± S.D. of three experiments. * *p* < 0.05; (**b**) The effects of compounds **1**–**3** on LPS-stimulated iNOS and COX-2 mRNA expression. The mRNA levels were examined by RT-PCR analysis. The relative intensity of iNOS/COX-2 to β-actin bands was measured by densitometry. The values represent the means ± S.D. of three experiments. * *p* < 0.05; (**c**) The effects of compounds **1**–**3** on LPS-induced I-κBα degradation and p65 level in nucleus of BV-2 microglia. Cells were treated with compounds **1**–**3** for 30 min prior to activation of LPS (0.1 μg/mL). After 15 min treatment of LPS, cytosolic I-κBα and nuclear p65 were analyzed by western blot. Images are the representative of three independent experiments that show similar results.

To reveal the underlying mechanism of compounds for the inhibition of NO and PGE_2_ production in LPS-treated microglia, protein and mRNA levels of iNOS and COX-2 were investigated by western blot and RT-PCR analysis. As shown in Figure 3a, compounds **1**–**3** (5 μM) suppressed the iNOS protein expression in BV-2 microglia cells, whereas LPS markedly enhanced the protein levels of iNOS. Compounds **1** and **2** also downregulated COX-2 protein expression compared with LPS control, while compound **3** was not effective. Furthermore, compounds **1**–**3** attenuated the iNOS and COX-2 mRNA levels whereas LPS treatment distinctly up-regulated their mRNA levels (Figure 3b). The suppressive effects of compounds **1**–**3** on iNOS expression level were not exactly parallel with the COX-2 level. These results suggest that compounds **1**–**3** inhibit the transcriptional expression of LPS-induced iNOS and COX-2.

Next we examined the effect of compounds **1**–**3** on nuclear factor κB (NF-κB) that regulates the expression of pro-inflammatory enzymes such as iNOS and COX-2 [26]. NF-κB, a transcription factor complex of p50 and p65 subunits, is found in cytoplasm as an inactive p50/p65 dimer. Inhibitor κB (I-κB) physically combines with p50/p65 dimer to suppress the NF-κB activation in normal conditions [27]. In response to pro-inflammatory stimuli, I-κB is rapidly degraded to release p50/p65. Free p50/p65 moves to the nucleus and induces the expression of pro-inflammatory genes [28]. To disclose the molecular mechanism for their inhibitory effect on pro-inflammatory enzyme expressions, we investigated whether compounds **1**–**3** affect the LPS-induced I-κBα degradation and nuclear level of p65 subunit of NF-κB. As shown in Figure 3c, compounds **1**–**3** suppressed the LPS-induced degradation of I-κBα and decreased the level of nuclear p65 by the treatment of **1**–**3** (at 5 μM). In addition, compound **1** with presence of non-cyclic prenyl moiety most potently suppressed degradation of I-κBα and nuclear level of NF-κB than those of cyclic pyranoprenyl chalcones **2** and **3**. Moreover, the results of isobavachalcone (**1**) showed concurrence with previous reports that showed its potentials for anti-inflammatory drug and therapeutic candidate against Parkinson’s disease by suppressing the TLR agonist induced-iNOS expression in RAW 264.7 macrophages and inhibiting the NF-κB activation in BV-2 microglia [29,30].

Taken together, these results demonstrate that prenyated chalcones **1**–**3** reduce the degradation of I-κBα and nuclear level of NF-κB in LPS-stimulated BV-2 microglia.

In summary, we isolated three prenylated chalcone derivatives from *Cullen corylifolium*— isobavachalcone (**1**), bavachromene (**2**) and kanzonol B (**3**)—as potent NO inhibitors in LPS-activated microglia. They also suppress PGE_2_ production and down-regulate the expression of iNOS and COX-2 through suppressing I-κBα degradation in LPS-activated microglia. These results imply that prenylated chalcones from *Cullen corylifolium* could be used for the treatment of neuro-inflammatory diseases.

## 3. Materials and Methods

### 3.1. General Information

1D and 2D NMR spectra were obtained on a VARIAN UNITY INOVA 400 spectrometer (Varian, Palo Alto, CA, USA). Mass spectra were determined on a JEOL JMS-AX505WA mass spectrometer (Tokyo, Japan). Column chromatography was carried out over silica gel (40–60 μm, Merck, Kenilworth, NJ, USA) and LiChroprep RP-C18 (40–60 μm, Merck). HPLC (high performance liquid chromatography) was carried out on a Waters 1525 system (Miliford, MA, USA) using reverse-phase column (ODS-2, 5 μm, 4.6 × 15 cm, GL Science, Seoul, Korea). Fractions obtained from column chromatography were monitored by thin layer chromatography (TLC) (RP-C18 F_254S_ and silica gel 60 F_254_, Merck).

### 3.2. Plant Material, Extraction and Isolation

The seeds of *Cullen corylifolium* (L.) Medik. (syn. *Psoralea corylifolia* L.) were purchased from the Kyungdong Hearbal Market in Seoul, Korea. Authentication of plant material was carried out by Prof. K.S. Yang at College of Pharmacy, Sookmyung Women’s University. A voucher specimen (No. SPH 13003) was deposited in the herbarium of Sookmyung Women’s University. The air-dried plant materials (8.8 kg) were washed with 10 L of *n*-hexane to remove fat. The remained materials were extracted with 10 L of methanol three times at room temperature for 24 h. The concentrated alcoholic extracts (1.6 kg) were suspended in water and successively partitioned with 20% hexane and EtOAc. The activity-guided chromatography for EtOAc soluble fraction was performed to purify the active principles. The EtOAc fraction (153.83 g) was subjected to silica gel column chromatography eluting with *n*-hexane:EtOAc gradient system (4:1 → 1:1) and 19 fractions were collected. Fraction 7 was further chromatographed on a RP-C18 column with a gradient elution of MeOH (50% → 100%) to afford 13 sub-fractions. Sub-fraction 7−13 was rechromatographed on silica gel with CH_2_Cl_2_:EtOAc isocratic system (100:1) to afford five sub-fractions. Each of subfraction 7-13-2 and 7-13-4 was further purified by a silica gel column with *n*-hexane:acetone gradient system (10:1 → 1:1) as eluents to yield compound **2** (17 mg) and compound **3** (6 mg). Fraction 12 was chromatographed on silica gel with *n*-hexane:EtOAc (contained 10% isopropanol) gradient system (20:1 → 1:3) to afford 14 subfractions. Subfraction 12-6 was rechromatographed on a silica gel column eluting with *n*-hexane:EtOAc (contained 10% isopropanol) gradient system (30:1 → 1:1) and then fraction 12-6-7 was further purified by a silica gel column with CHCl_3_:MeOH gradient system (100:1 → 20:1) to yield compound **1** (12 mg). The purity of compounds was confirmed by HPLC analysis and their ^1^H-NMR spectra. The structures of compounds were elucidated by the analysis of their corresponding mass and NMR spectroscopic data (Supplementary Materials, Figures S1–S15).

*Isobavachalcone* (**1**).^1^H-NMR (CD_3_OD, 400 MHz) δ: 7.82 (1H, d, *J* = 9.2, H-6′), 7.77 (1H, d, *J* = 15.6, H-β), 7.61 (1H, d, *J* = 15.6, H-α), 7.60 (2H, d, *J* = 8.4, H-2, H-6), 6.84 (2H, d, *J* = 8.4, H-3, H-5), 6.43 (1H, d, *J* = 9.2, H-5′), 5.23 (1H, m, H-2′′), 3.32 (1H, m, H-1′′), 1.78 (3H, s, H-4′′), 1.66 (3H, s, H-5′′). ^13^C-NMR (CD_3_OD, 100 MHz) δ: 193.7 (C=O), 165.2 (C-4′), 163.6 (C-2′), 161.5 (C-4), 145.3 (C-β), 131.9 (C-3′′), 131.7 (C-2), 131.7 (C-6), 130.4 (C-6′), 123.6 (C-2′′), 118.6 (C-α), 116.9 (C-3), 116.9 (C-5), 116.6 (C-3′), 114.5 (C-1), 114.5 (C-1′) 108.2 (C-5′), 26.0 (C-5′′), 22.5 (C-1′′), 17.9 (C-4′′). FABMS: *m*/*z* 325 [M + 1]^+^.

*Bavachromene* (**2**). ^1^H-NMR (CD_3_OD + CDCl_3_, 400 MHz) δ: 7.77 (1H, d, *J* = 15.2, H-β), 7.58 (1H, s, H-6′), 7.55 (2H, d, *J* = 8.8, H-2, H-6), 7.45 (1H, d, *J* = 15.2, H-α), 6.84 (2H, d, *J* = 8.8, H-3, H-5), 6.34 (1H, d, *J* = 10.0, H-10′), 6.28 (1H, s, H-3′), 5.59 (1H, d, *J* = 10.0, H-9′), 1.42 (6H, s, H-11′, H-12′). ^13^C-NMR (CD_3_OD + CDCl_3_, 100 MHz) δ: 192.8 (C=O), 166.4 (C-4′), 161.0 (C-2′), 160.7 (C-4), 145.6 (C-β), 131.3 (C-2, C-6), 129.3 (C-9′), 128.3 (C-6′), 127.0 (C-1), 121.6 (C-10′), 117.5 (C-α), 116.5 (C-3, C-5), 114.8 (C-1′), 114.3 (C-5′), 104.8 (C-3′), 78.6 (C-8′) 28.8 (C-11′, C-12′). FABMS: *m*/*z* 323 [M + 1]^+^.

*Kanzonol B* (**3**). ^1^H-NMR (CD_3_OD + CDCl_3_, 400 MHz) δ: 7.84 (1H, d, *J* = 8.8, H-6′), 7.73 (1H, d, *J* = 15.2, H-β), 7.48 (1H, d, *J* = 15.2, H-α), 7.43 (1H, dd, *J* = 2.0, 8.4, H-2), 7.31 (1H, d, *J* = 2.4, H-6), 6.77 (1H, d, *J* = 8.4, H-3), 6.41 (1H, dd, *J* = 2.4, 8.8, H-5′), 6.37 (1H, d, *J* = 9.6, H-10), 6.32 (1H, d, *J* = 2.4, H-3′), 5.69 (1H, d, *J* = 9.6, H-9), 1.43 (6H, s, H-11, H-12). ^13^C-NMR (CD_3_OD + CDCl_3_, 100 MHz) δ: 192.7 (C=O), 166.6 (C-2′), 165.5 (C-4′), 156.2 (C-4), 144.7 (C-β), 132.6 (C-6′), 132.0 (C-9), 130.7 (C-2), 128.3 (C-1), 127.2 (C-6), 122.2 (C-10), 122.1 (C-5), 118.4 (C-α), 117.5 (C-3), 114.1 (C-1′), 108.8 (C-5′), 103.6 (C-3′), 77.8 (C-8), 28.4 (C-11, C-12). FABMS: *m*/*z* 323 [M + 1]^+^.

### 3.3. Cell Culture

BV-2 microglial cells (ATCC, Rockville, MD, USA) were grown in DMEM supplemented with 10% FBS, 100 units/mL penicillin, and 100 μg/mL streptomycin (Life Technologies, Frederick, MD, USA). Cells were maintained at 37 °C with 5% CO_2_ in a humidified atmosphere. All test concentrations of compounds showed no significant toxicity. The cell viability was determined by MTT assay.

### 3.4. Measurement of Nitric Oxide Production

BV-2 microglial cells (1 × 10^5^ cells/mL in 48-well plate) were treated with LPS (0.1 μg/mL) in the absence or presence of test compounds for 20 h. NO was measured by detecting the accumulated nitrite by Griess method. Briefly, samples (100 μL) of culture media were combined with 150 μL of Griess reagent (1% sulfanilamide, 0.1% naphthylethylene diamine in 2.5% phosphoric acid solution) and then incubated at room temperature for 10 min. Absorbance was detected at 540 nm by using a microplate reader (Molecular Devices, Sunnyvale, CA, USA). The concentration of NO was determined by the sodium nitrite standard curve.

### 3.5. Peroxynitrite (ONOO^−^) Scavenging Assay

10 μL of test compounds were added to the reaction buffer containing 5 μM dihydrorhodamine 123 (DHR 123, Molecular Probes, Eugene, OR, USA) and 100 μM diethylenetriaminepentaacetic acid (DTPA). After the treatment with presence or absence (background) of native 10 μM ONOO^−^ (Cayman Chemical Co., Ann Arbor, MI, USA) for 5 min, the final and background fluorescent intensities were measured. Penicillamine was used as a positive control. The fluorescence was measured at room temperature by a microplate fluorescence spectrophotometer (Spectra Max Gemini XS, Molecular Devices, Sunnyvale, CA, USA) with excitation and emission wavelength of 500 and 530 nm, respectively. ONOO^−^ scavenging activity (%) of test compounds were expressed as the ratio of the decreased fluorescence to the control fluorescence. The concentration of ONOO^−^ solution was determined spectrophotometrically (ε_302_ = 1670 M^−1^ cm^−1^) using Ultraspec 2000 UV/visible spectrophotometer (Pharmacia-Biotech, Cambridge, UK).

### 3.6. Prostagandin E2 Assay

To examine the effects of compounds on COX-2, cells were attached with aspirin (500 μM) to inactivate the COX-1. After 2 h, cells were washed with fresh media three times and incubated with LPS (0.1 μg/mL) in presence of compounds for 20 h. The PGE_2_ levels in culture media were determined using enzyme immunoassay kit (Cayman Chemical) according to the manufacturer’s instruction. Briefly, 50 μL of supernatant of the culture medium and 50 μL PGE_2_ tracer were put into the PGE_2_ EIA plate and incubated for 18 h at room temperature. The wells were washed with 10 mM phosphate buffer (pH 7.4) containing 0.05% Tween 20. Then 200 μL of Ellman’s reagent was added to the well and incubated in the dark. Following the developing step, the absorbance was read at 405 nm by a microplate reader. A standard curve was prepared simultaneously with PGE_2_ standard ranging from 0.05 to 6 ng/mL.

### 3.7. Western Blot Analysis

BV-2 microglia (5 × 10^5^ cells/60 mm dish) were treated with LPS (0.1 μg/mL) in the absence or presence of test compounds. After 20 h treatment, cells were lysed with cell lysis buffer (Cell Signaling Technologies, Beverly, MA, USA) and centrifuged at 4 °C. Supernatant were collected and protein concentrations were determined by the Bradford method. For preparation of cytosol and nuclear extracts, cells were treated with test compounds for 30 min prior to the stimulation with 0.1 μg/mL LPS. Following 15 min treatment of LPS, cells were collected by using NE-PER nuclear and cytoplasmic extraction reagents according to the manufacturer’s instructions (Pierce Biotechnology, Rockford, IL, USA). For immunoblot analysis, antibodies against iNOS, COX-2, I-κBα and p65 were obtained from BD Biosciences (Franklin Lakes, NJ, USA), Cayman Chemical Company and Santa Cruz Biotechnology (Rockford, IL, USA), respectively.

### 3.8. Reverse Transcription and Polymerase Chain Reaction (RT-PCR) Analysis

BV-2 microglia (5 × 10^5^ cells/60 mm dish) were incubated with or without test compounds in presence of LPS (0.1 μg/mL). Total RNA was isolated from the cells by TRIzol (Life Technologies) in accordance with the manufacturer’s instructions, and then reverse transcribed into cDNA by using reverse transcriptase (Life Technologies). The cDNA amplification was carried out to detect the target genes (iNOS, COX-2, and β-actin) by using a recombinant Taq polymerase (Promega, Madison, WI, USA).

### 3.9. Statistical Anaylsis

The results were expressed as mean ± S.D. of three experiments, and statistical analysis was performed using one way analysis of variance (ANOVA) and Student’s *t*-test. A *p* value of <0.05 was regarded as significantly different.

## Figures and Tables

**Figure 1:**
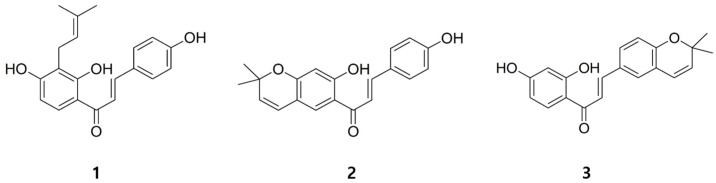
The chemical structures of compounds **1**–**3** from *Cullen corylifolium*.

## Supplementary Materials

The supplementary materials are available online. ^1^H-NMR, ^13^C-NMR, COSY, HSQC and HMBC spectra of compounds **1**, **2** and **3** are available as supporting information.

## References

[1] Dheen S.T., Kaur C., Ling E.A. (2007). Microglial activation and its implications in the brain diseases. Curr. Med. Chem..

[2] Calabrese V., Boyd-Kimball D., Scapagnini G., Butterfield D.A. (2004). Nitric oxide and cellular stress response in brain aging and neurodegenerative disorders: The role of vitagenes. In Vivo.

[3] Blaylock R.L. (2017). Parkinson’s disease: Microglial/macrophage-induced immunoexcitotoxicity as a central mechanism of neurodegeneration. Surg. Neurol. Int..

[4] Forstermann U., Schmidt H.H., Pollock J.S., Sheng H., Mitchell J.A., Warner T.D., Nakane M., Murad F. (1991). Isoforms of nitric oxide synthase. Characterization and purification from different cell types. Biochem. Pharmacol..

[5] Whittle B.J. (1995). Nitric oxide in physiology and pathology. Histochem. J..

[6] Gebicke-Haerter P.J. (2001). Microglia in neurodegeneration: Molecular aspects. Microsc. Res. Tech..

[7] Ivanov A.I., Romanovsky A.A. (2004). Prostaglandin E2 as a mediator of fever: Synthesis and catabolism. Front. Biosci..

[8] Sales K.J., Jabbour H.N. (2003). Cyclooxygenase enzymes and prostaglandins in pathology of the endometrium. Reproduction.

[9] Johnson R.W. (2015). Feeding the beast: Can microglia in the senesent brain be regulated by diet?. Brain Behav. Immun..

[10] Wang Y., Plastina P., Vincken J.P., Jansen R., Balvers M., Ten Klooster J.P., Gruppen H., Witkamp R., Meijerink J. (2017). N-docosahexaenoyl dopamine, an endocannabinoid-like conjugate of dopamine and the n-3 fatty acid docosahexaenoic acid, attenuates lipopolysaccharide-induced activation of microglia and macrophages via COX-2. ACS Chem. Neurosci..

[11] Chopra B., Dhingra A.K., Dhar K.L. (2013). *Psoralea corylifolia* L. (Buguchi)—Folklore to modern evidence: Review. Fitoterapia.

[12] Lee M.H., Kim J.Y., Ryu J.H. (2005). Prenylflavones from *Psoralea corylifolia* inhibit nitric oxide synthase expression through the inhibition of I-kappaB-alpha degradation in activated microglial cells. Biol. Pharm. Bull..

[13] Bronikowska J., Szliszka E., Jaworska D., Czuba Z.P., Krol W. (2012). The coumarin psoralidin enhances anticancer effect of tumor necrosis factor-related apoptosis-inducing ligand (TRAIL). Molecules.

[14] Srinivasan S., Sarada D.V. (2012). Antifungal activity of phenyl derivative of pyranocoumarin from *Psoralea corylifolia* L. seeds by inhibition of acetylation activity of trichothecene 3-o-acetyltransferase (Tri101). J. Biomed. Biotechnol..

[15] Wu C.Z., Hong S.S., Cai X.F., Dat N.T., Nan J.X., Hwang B.Y., Lee J.J., Lee D. (2008). Hypoxia-inducible factor-1 and nuclear factor-kappaB inhibitory meroterpene analogues of bakuchiol, a constituent of the seeds of *Psoralea corylifolia*. Bioorg. Med. Chem. Lett..

[16] Xin D., Wang H., Yang J., Su Y.F., Fan G.W., Wang Y.F., Zhu Y., Gao X.M. (2010). Phytoestrogens from *Psoralea corylifolia* reveal estrogen receptor-subtype selectivity. Phytomedicine.

[17] Park J., Kim D.H., Ahn H.N., Song Y.S., Lee Y.J., Ryu J.H. (2012). Activation of Estrogen Receptor by Bavachin from *Psoralea corylifolia*. Biomol. Ther..

[18] Pistelli L., Spera K., Flamini G., Mele S., Morelli I. (1996). Isoflavonoids and chalcones from *Anthyllis hermanniae*. Phytochemistry.

[19] Haraguchi H., Inoue J., Tamura Y., Mizutani K. (2002). Antioxidative components of *Psoralea corylifolia* (Leguminosae). Phytother. Res..

[20] Kuete V., Sandjo L.P. (2012). Isobavachalcone: An overview. Chin. J. Integr. Med..

[21] Gupta B.K., Gupta G.K., Dhar K.L., Atal C.K. (1980). A C-formylated chalcone from *Psoralea corylifolia*. Phytochemistry.

[22] Thoshio F., Junko N., Taro N. (1993). Five isoprenoid-substituted flavonoids from *Glycyrrhiza eurycarpa*. Phytochemistry.

[23] Beckman J.S., Beckman T.W., Chen J., Marshall P.A., Freeman B.A. (1990). Apparent hydroxyl radical production by peroxynitrite: Implications for endothelial injury from nitric oxide and superoxide. Proc. Natl. Acad. Sci. USA.

[24] Guix F.X., Wahle T., Vennekens K., Snellinx A., Chavez-Gutierrez L., Ill-Raga G., Ramos-Fernandez E., Guardia-Laguarta C., Lleo A., Arimon M., Berezovska O. (2012). Modification of gamma-secretase by nitrosative stress links neuronal ageing to sporadic Alzheimer’s disease. EMBO Mol. Med..

[25] Lee M.H., Kim J.Y., Yoon J.H., Lim H.J., Kim T.H., Jin C., Kwak W.J., Han C.K., Ryu J.H. (2006). Inhibition of nitric oxide synthase expression in activated microglia and peroxynitrite scavenging activity by *Opuntia ficus indica* var. *saboten*. Phytother. Res..

[26] Ahn K.S., Aggarwal B.B. (2005). Transcription factor NF-kappaB: A sensor for smoke and stress signals. Ann. N. Y. Acad. Sci..

[27] Yamamoto M., Takeda K. (2008). Role of nuclear IkappaB proteins in the regulation of host immune responses. J. Infect. Chemother..

[28] Wan F., Lenardo M.J. (2010). The nuclear signaling of NF-kappaB: Current knowledge, new insights, and future perspectives. Cell Res..

[29] Jing H., Wang S., Wang M., Fu W., Zhang C., Xu D. (2017). Isobavachalcone attenuates MPTP-induced Parkinson’s disease in mice by inhibition of microglial activation through NF-kB pathway. PLoS ONE.

[30] Shin H.J., Shon D.H., Youn H.S. (2013). Isobavachalcone suppresses expression of inducible nitric oxide synthase induced by Toll-like receptor agonists. Int. Immunopharmacol..

