# Supplementary material for: Modulation of Inducible Nitric Oxide Synthase Expression in LPS-Stimulated BV-2 Microglia by Prenylated Chalcones from Cullen corylifolium (L.) Medik. through Inhibition of I-κBα Degradation
Source: Molecules. 2018 Jan 4;23(1):109. doi: 10.3390/molecules23010109 (PMC6017879; doi:10.3390/molecules23010109)
Supplement: Supplementary file 1 [file molecules-23-00109-s001.zip › Supplementary materials-proof reading-Ryu.pdf]

Supplementary Materials

Spectroscopic Analysis (NMR) of compounds 1 - 3

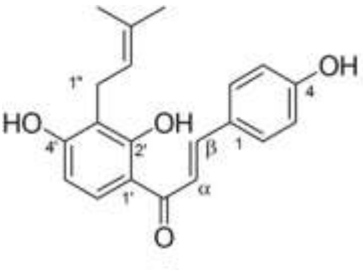

1

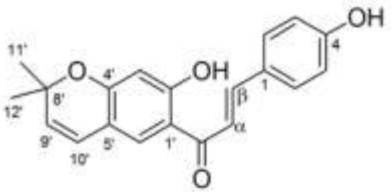

2

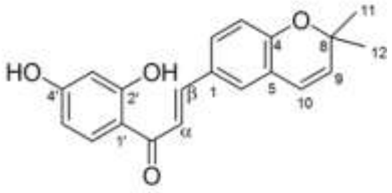

3

Compound 1 (isobavachalcone)

^{1}H NMR (CD_3OD, 400 MHz) δ: 7.82 (1H, d, *J* = 9.2, H-6′), 7.77 (1H, d, *J* = 15.6, H-β), 7.61 (1H, d, *J* = 15.6, H-α), 7.60 (2H, d, *J* = 8.4, H-2, H-6), 6.84 (2H, d, *J* = 8.4, H-3, H-5), 6.43 (1H, d, *J* = 9.2, H-5′), 5.23 (1H, m, H-2″), 3.32 (1H, m, H-1″), 1.78 (3H, s, H-4″), 1.66 (3H, s, H-5″).

^{13}C NMR (CD_3OD, 100 MHz) δ: 193.7 (C=O), 165.2 (C-4′), 163.6 (C-2′), 161.5 (C-4), 145.3 (C-β), 131.9 (C-3″), 131.7 (C-2), 131.7 (C-6), 130.4 (C-6′), 123.6 (C-2″), 118.6 (C-α), 116.9 (C-3), 116.9 (C-5), 116.6 (C-3′), 114.5 (C-1), 114.5 (C-1′) 108.2 (C-5′), 26.0 (C-5″), 22.5 (C-1″), 17.9 (C-4″).

Compound 2 (Bavachromene)

^{1}H NMR (CD_3OD + $CDCl_3$, 400 MHz) δ: 7.77 (1H, d, *J* =15.2, H-β), 7.58 (1H, s, H-6′), 7.55 (2H, d, *J* = 8.8, H-2, H-6), 7.45 (1H, d, *J* = 15.2, H-α), 6.84 (2H, d, *J* = 8.8, H-3, H-5), 6.34 (1H, d, *J* = 10.0, H-10′), 6.28 (1H, s, H-3′), 5.59 (1H, d, *J* = 10.0, H-9′), 1.42 (6H, s, H-11′, H-12′).

^{13}C NMR (CD_3OD + $CDCl_3$, 100 MHz) δ: 192.8 (C=O), 166.4 (C-4′), 161.0 (C-2′), 160.7 (C-4), 145.6 (C-β), 131.3 (C-2, C-6), 129.3 (C-9′), 128.3 (C-6′), 127.0 (C-1), 121.6 (C-10′), 117.5 (C- α), 116.5 (C-3, C-5), 114.8 (C-1′), 114.3 (C-5′), 104.8 (C-3′), 78.6 (C-8′) 28.8 (C-11′, C-12′).

Compound 3 (kanzonol B)

^{1}H NMR (CD_3OD + $CDCl_3$, 400 MHz) δ: 7.84 (1H, d, *J* = 8.8, H-6′), 7.73 (1H, d, *J* = 15.2, H-β), 7.48 (1H, d, *J* = 15.2, H-α), 7.43 (1H, dd, *J* = 2.0, 8.4, H-2), 7.31 (1H, d, *J* = 2.4, H-6), 6.77 (1H, d, *J* = 8.4, H-3), 6.41 (1H, dd, *J* = 2.4, 8.8, H-5′), 6.37 (1H, d, *J* = 9.6, H-10), 6.32 (1H, d, *J* = 2.4, H-3′), 5.69 (1H, d, *J* = 9.6, H-9), 1.43 (6H, s, H-11, H-12).

^{13}C NMR (CD_3OD + $CDCl_3$, 100 MHz) δ: 192.7 (C=O), 166.6 (C-2′), 165.5 (C-4′), 156.2 (C-4), 144.7 (C-β), 132.6 (C-6′), 132.0 (C-9), 130.7 (C-2), 128.3 (C-1), 127.2 (C-6), 122.2 (C-10), 122.1 (C-5), 118.4 (C- α), 117.5 (C-3), 114.1 (C-1′), 108.8 (C-5′), 103.6 (C-3′), 77.8 (C-8), 28.4 (C-11, C-12).

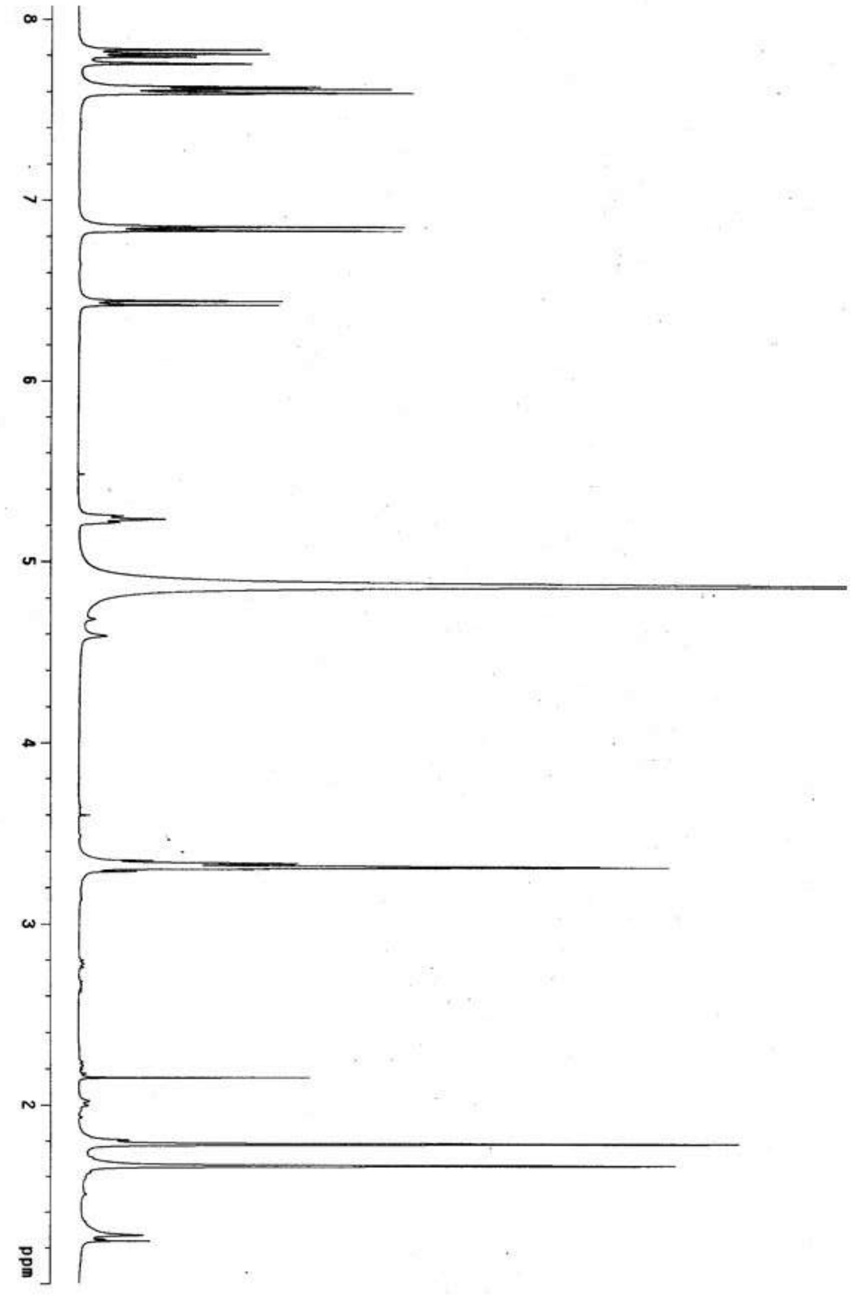

Figure S1. ^{1}H NMR spectrum of compound **1** (CD_3OD, 400MHz)

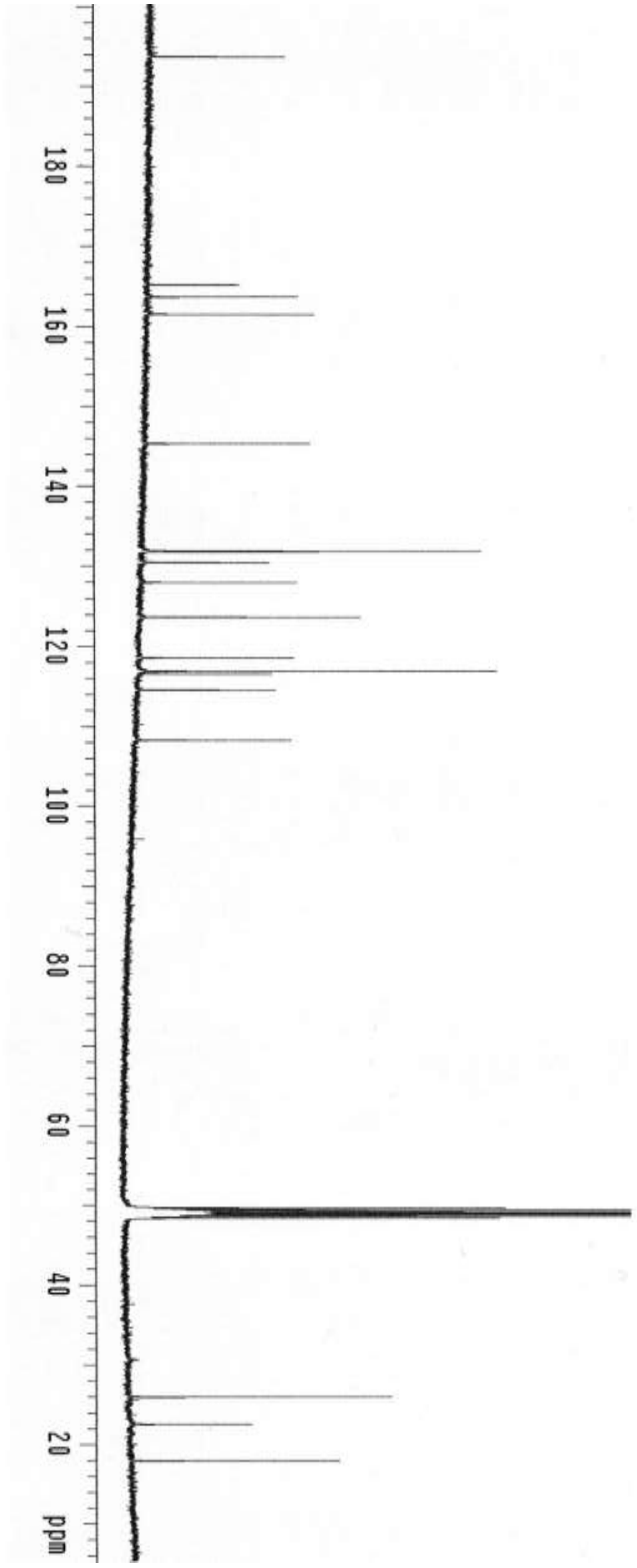

Figure S2. ^{13}C NMR spectrum of compound **1** (CD_3OD, 100MHz)

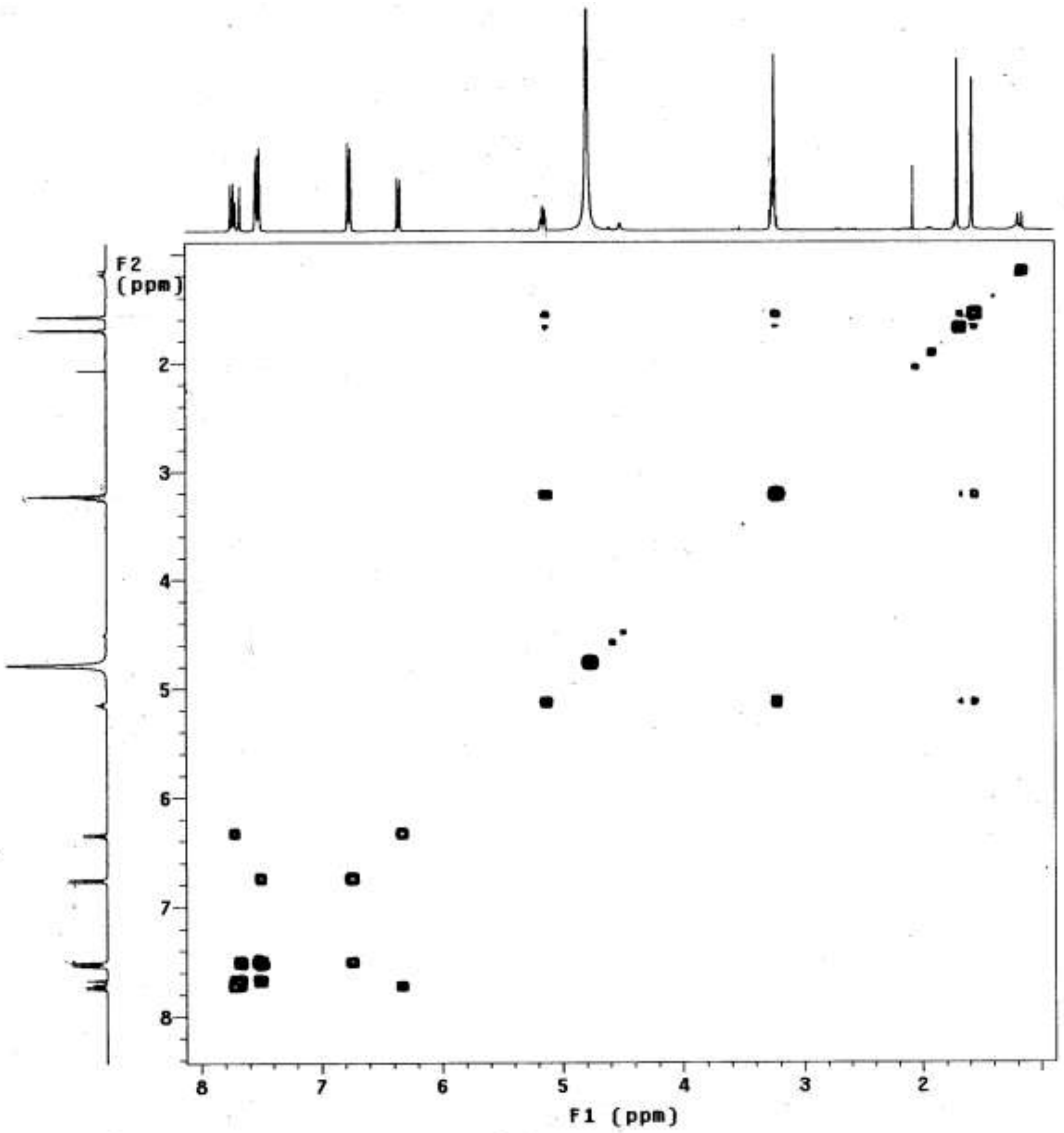

Figure S3. ^{1}H-^{1}H COSY spectrum of compound **1** (CD_3OD, 400MHz)

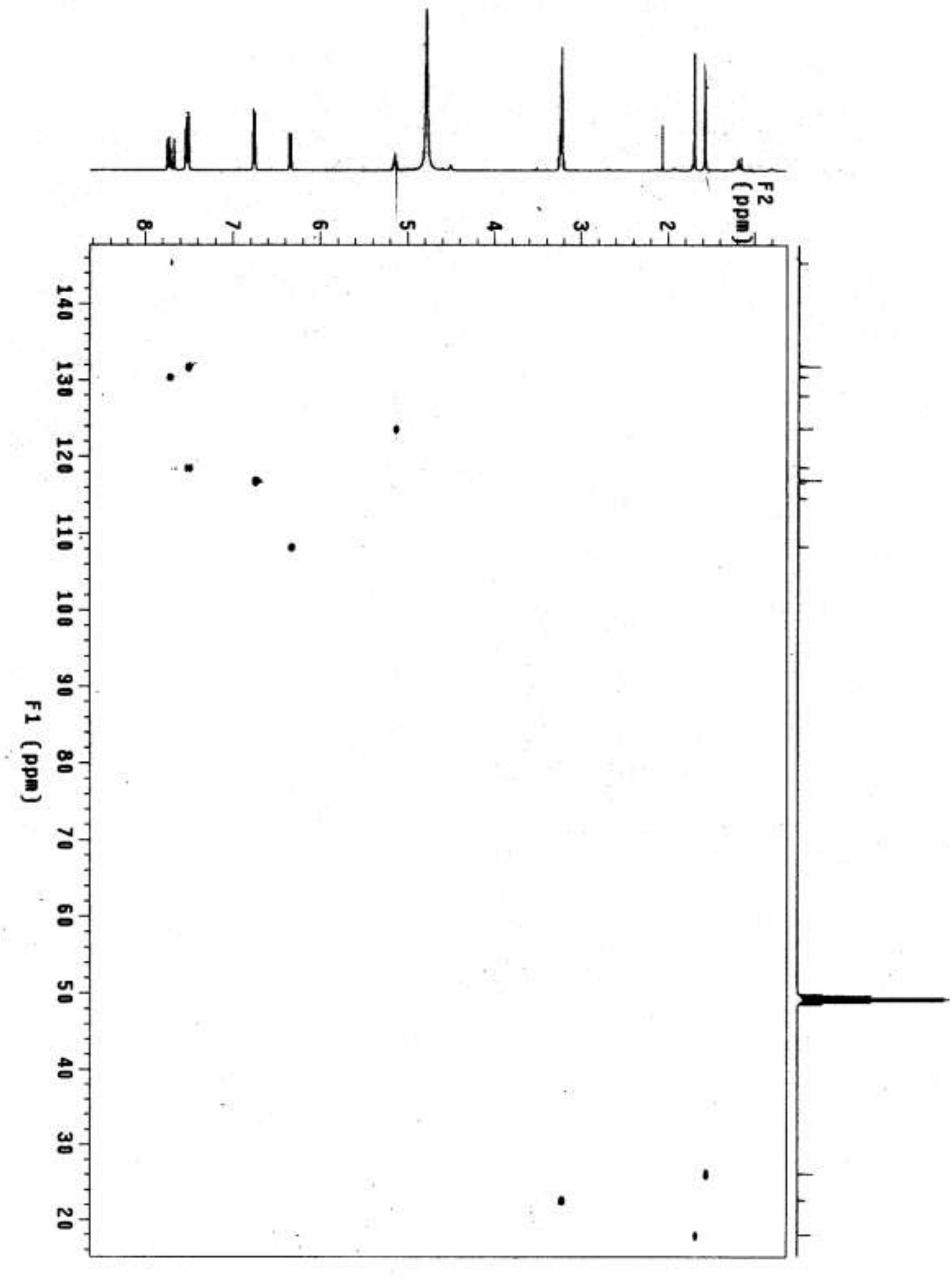

Figure S4. HSQC spectrum of compound **1** (CD_3OD, 400MHz)

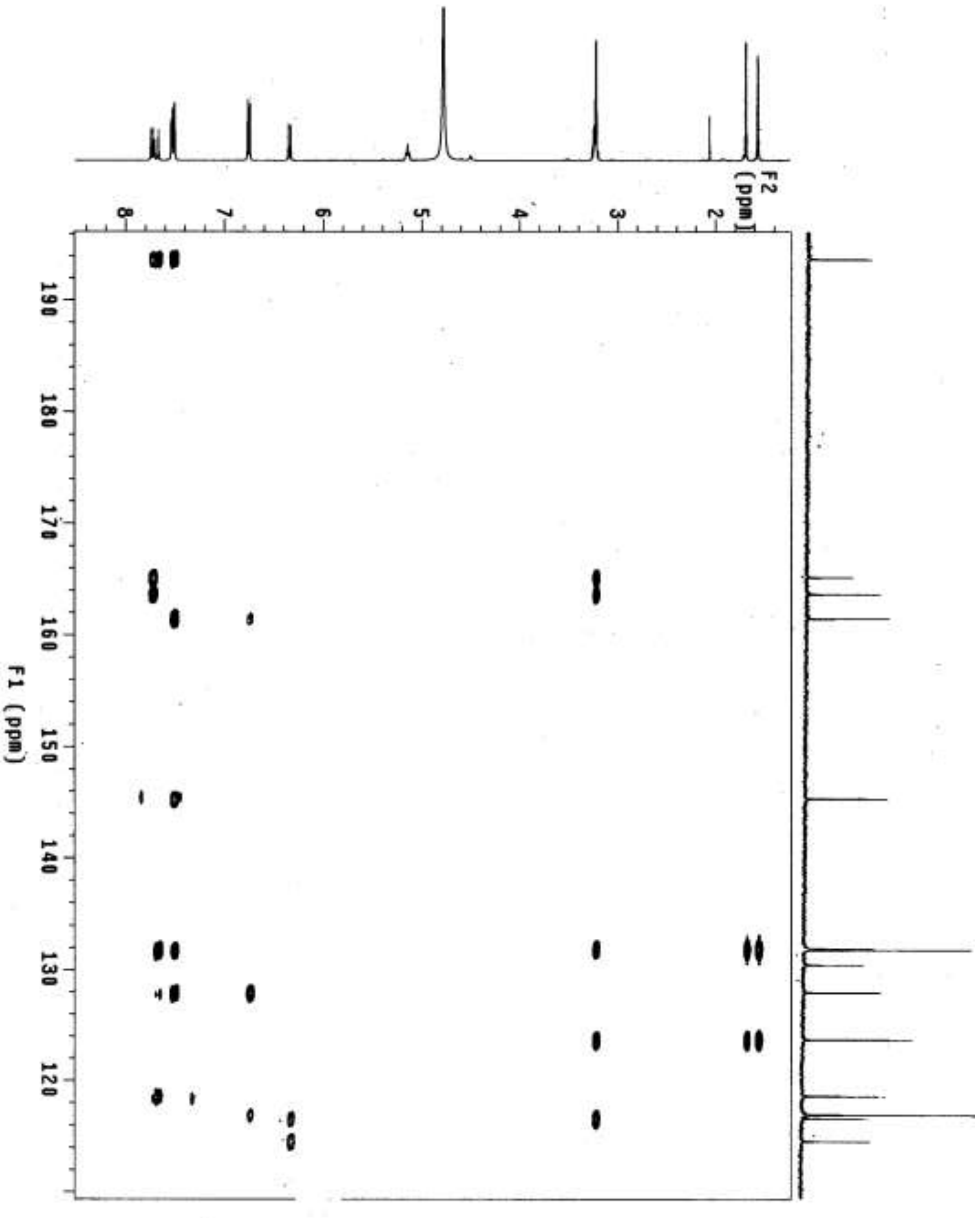

Figure S5. HMBC spectrum of compound **1** (CD_3OD, 400MHz)

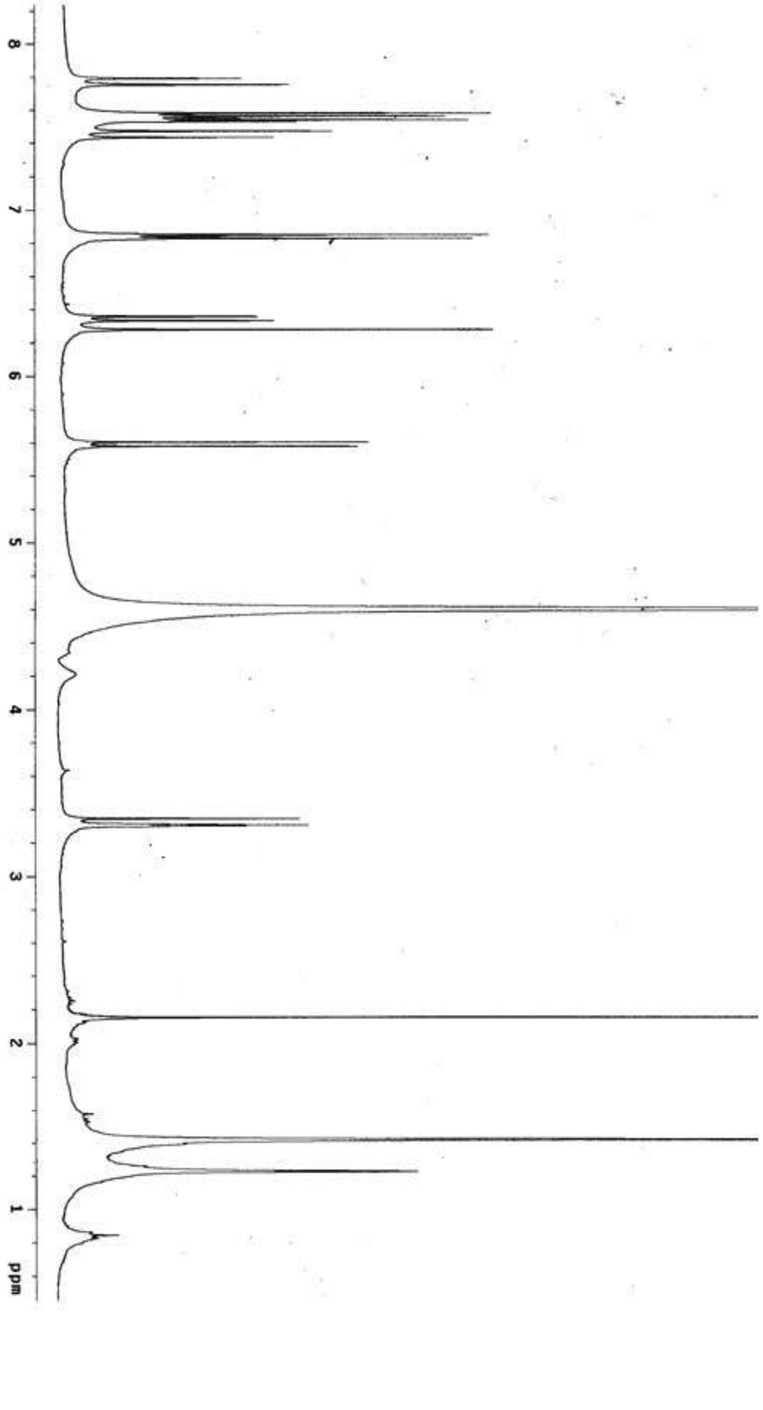

Figure S6. ^{1}H NMR spectrum of compound **2** (CD_3OD + $CDCl_3$, 400MHz)

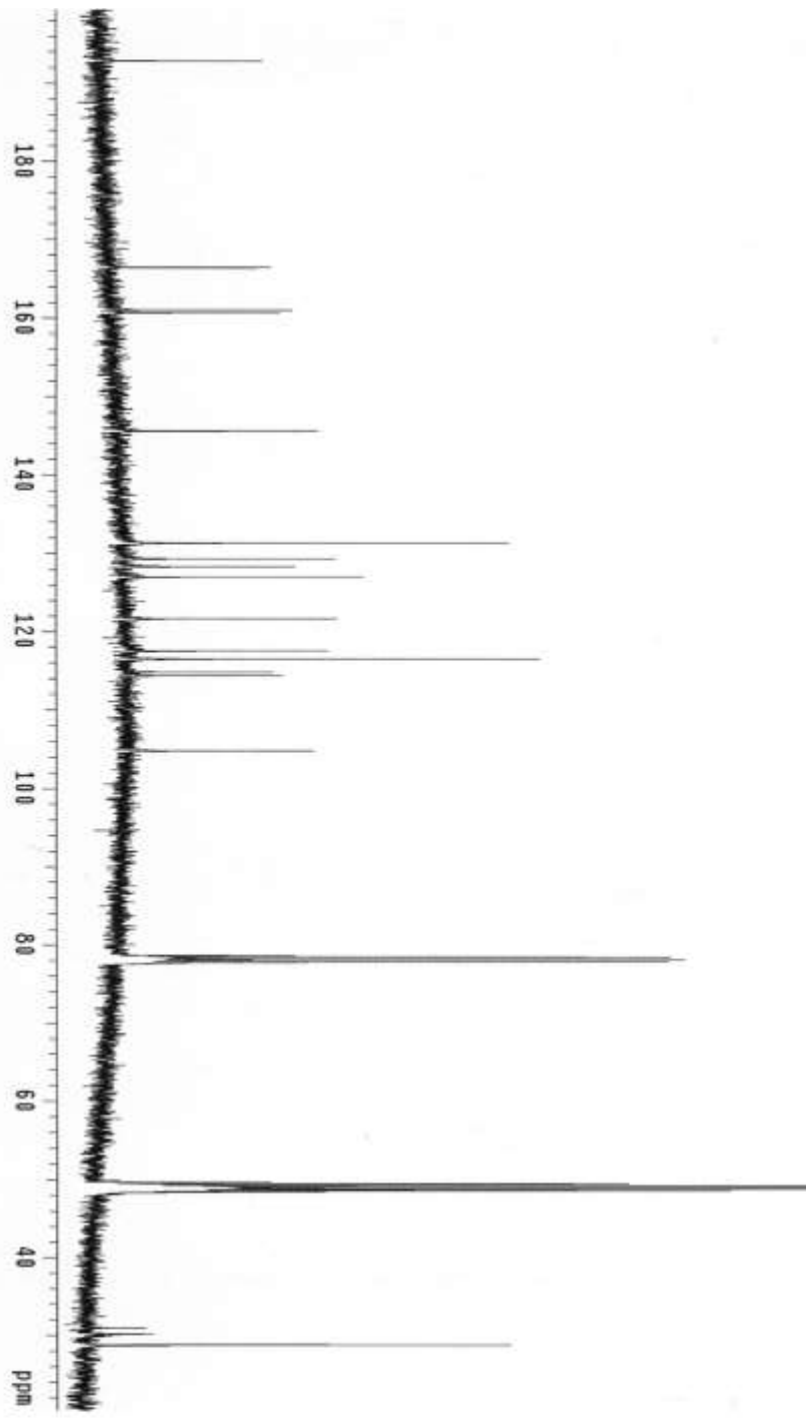

Figure S7. ^{13}C NMR spectrum of compound **2** (CD_3OD + $CDCl_3$, 100MHz)

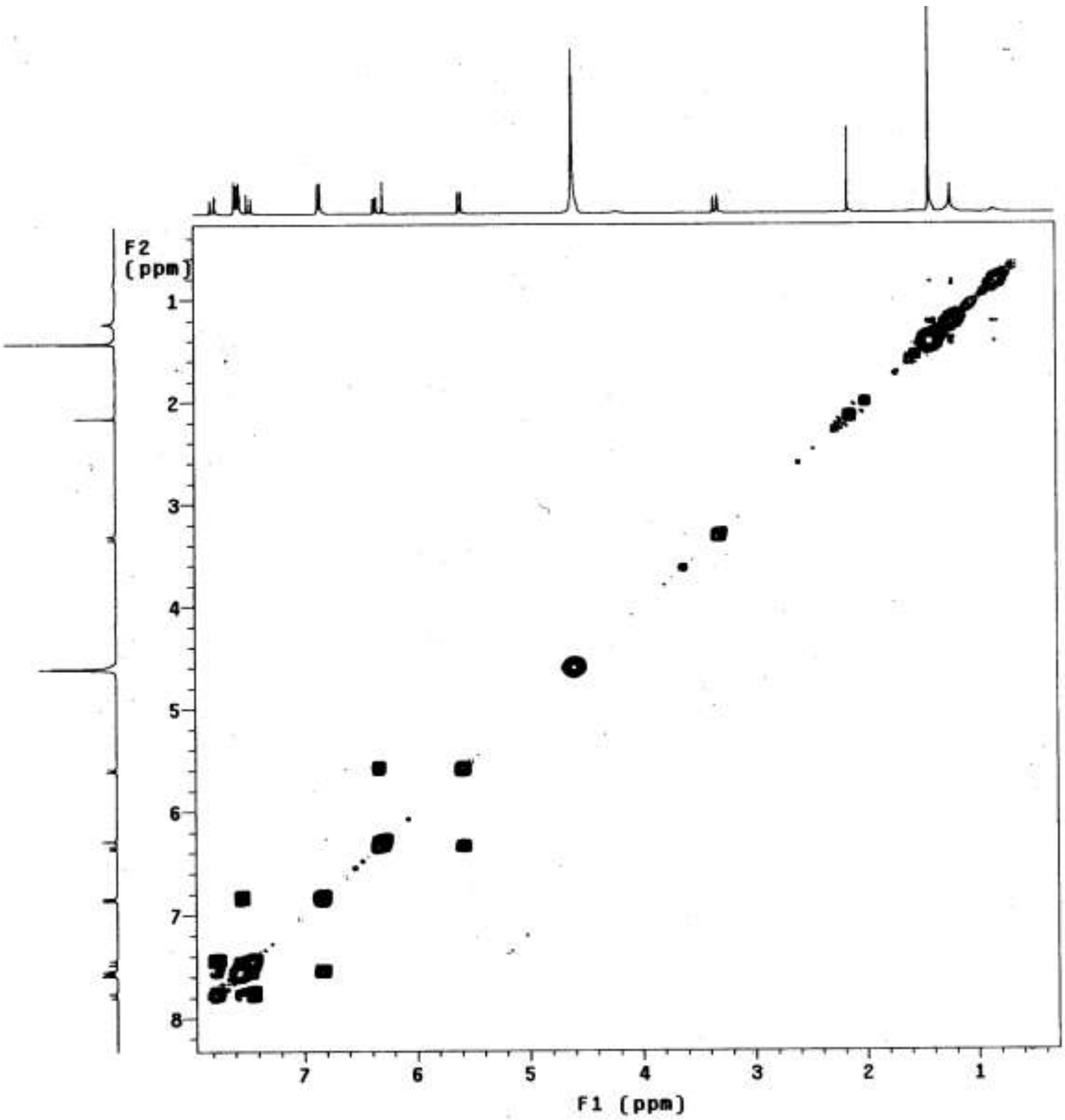

Figure S8. ^{1}H-^{1}H COSY spectrum of compound **2** (CD_3OD + $CDCl_3$, 400MHz)

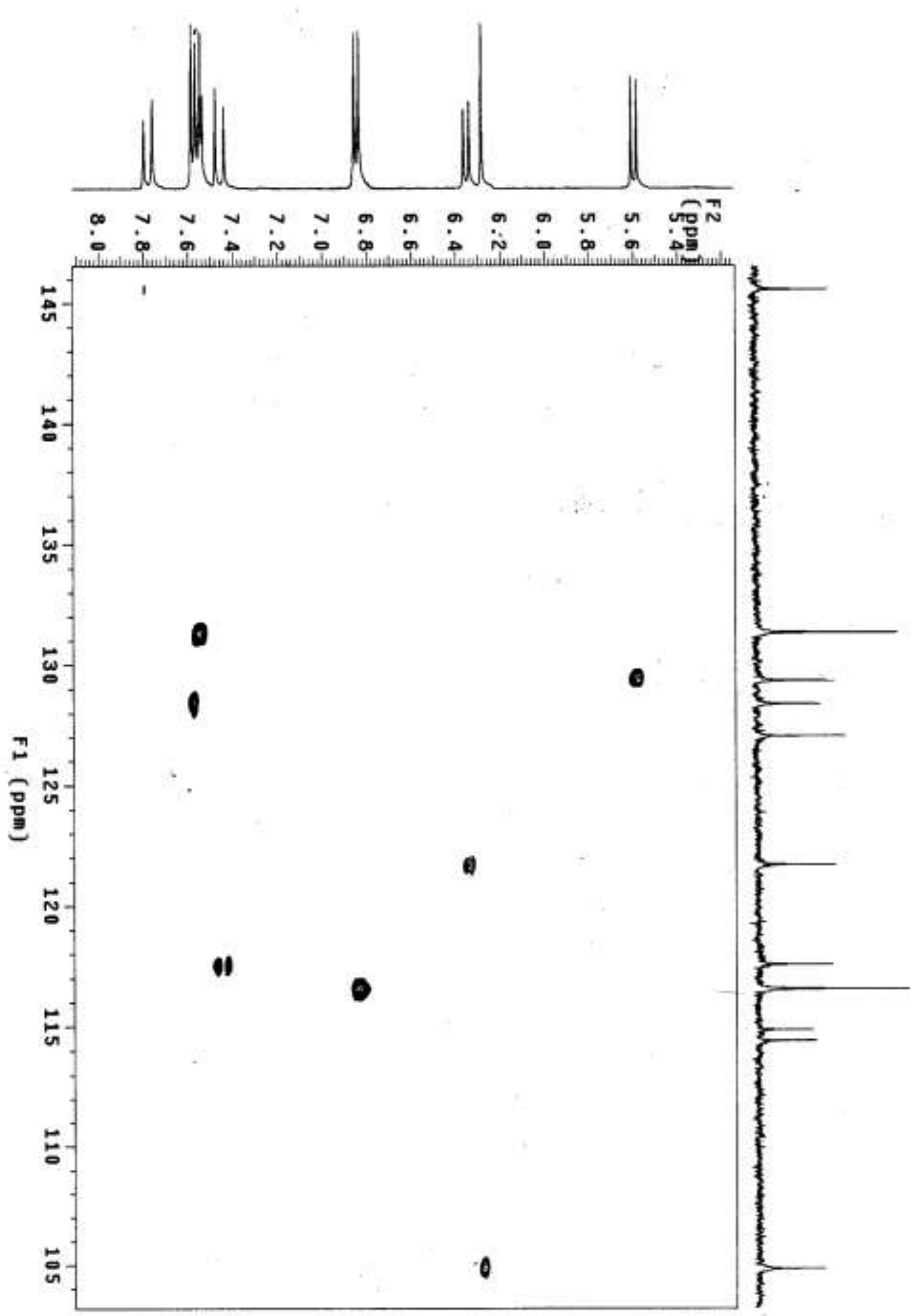

Figure S9. HSQC spectrum of compound **2** (CD_3OD + $CDCl_3$, 400MHz)

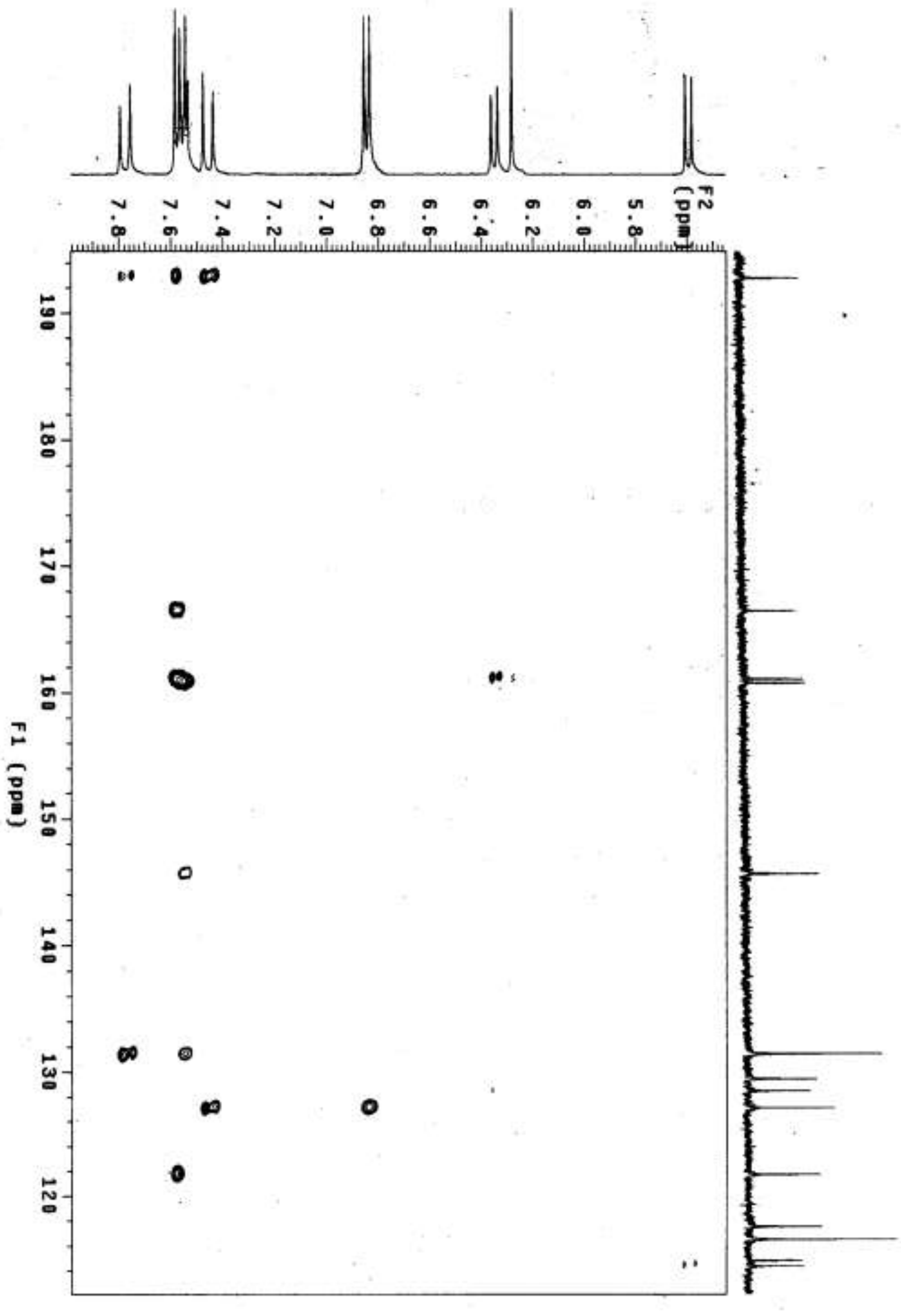

Figure S10. HMBC spectrum of compound **2** (CD_3OD + $CDCl_3$, 400MHz)

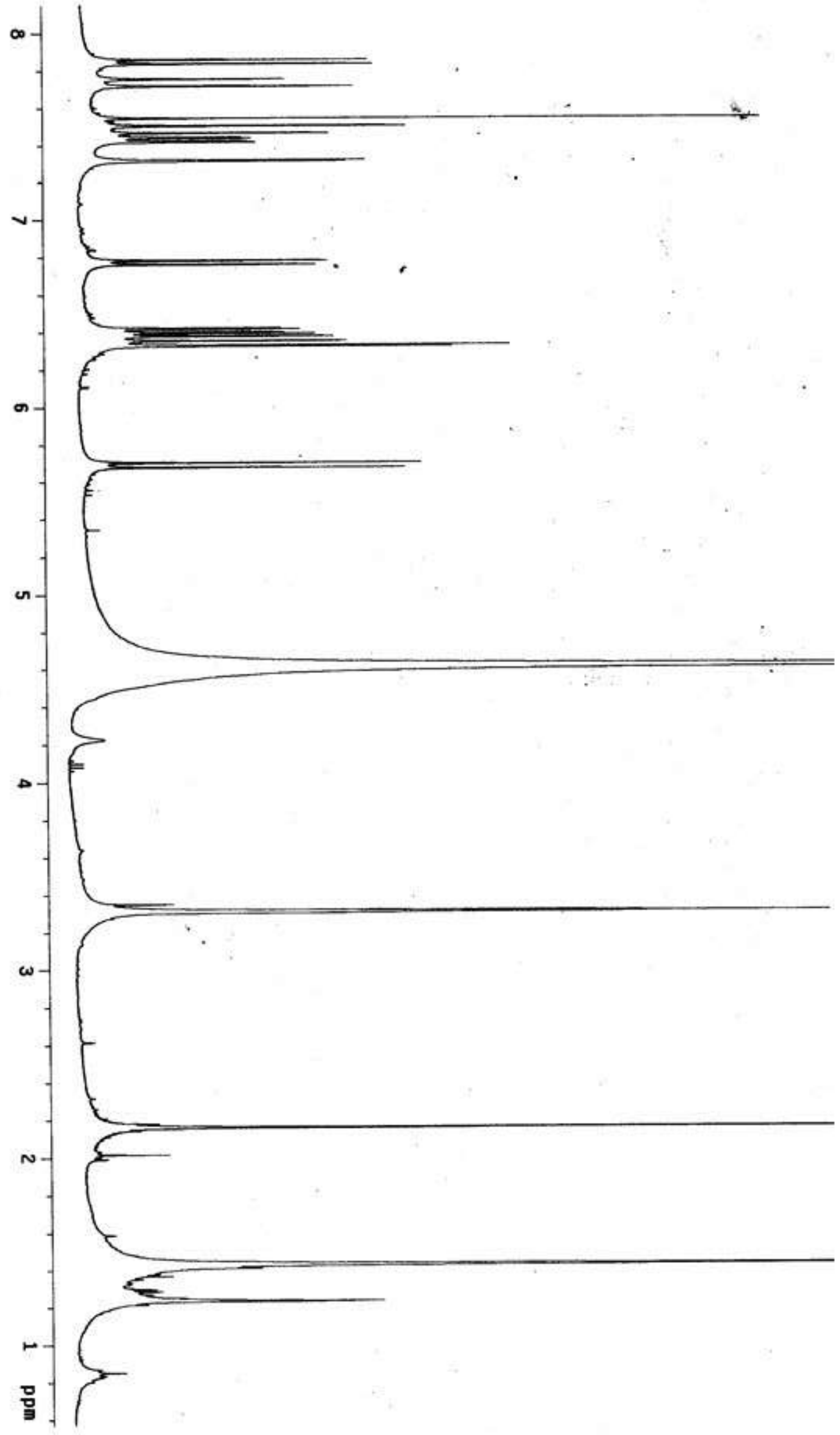

Figure S11. ^{1}H NMR spectrum of compound **3** (CD_3OD + $CDCl_3$, 400MHz)

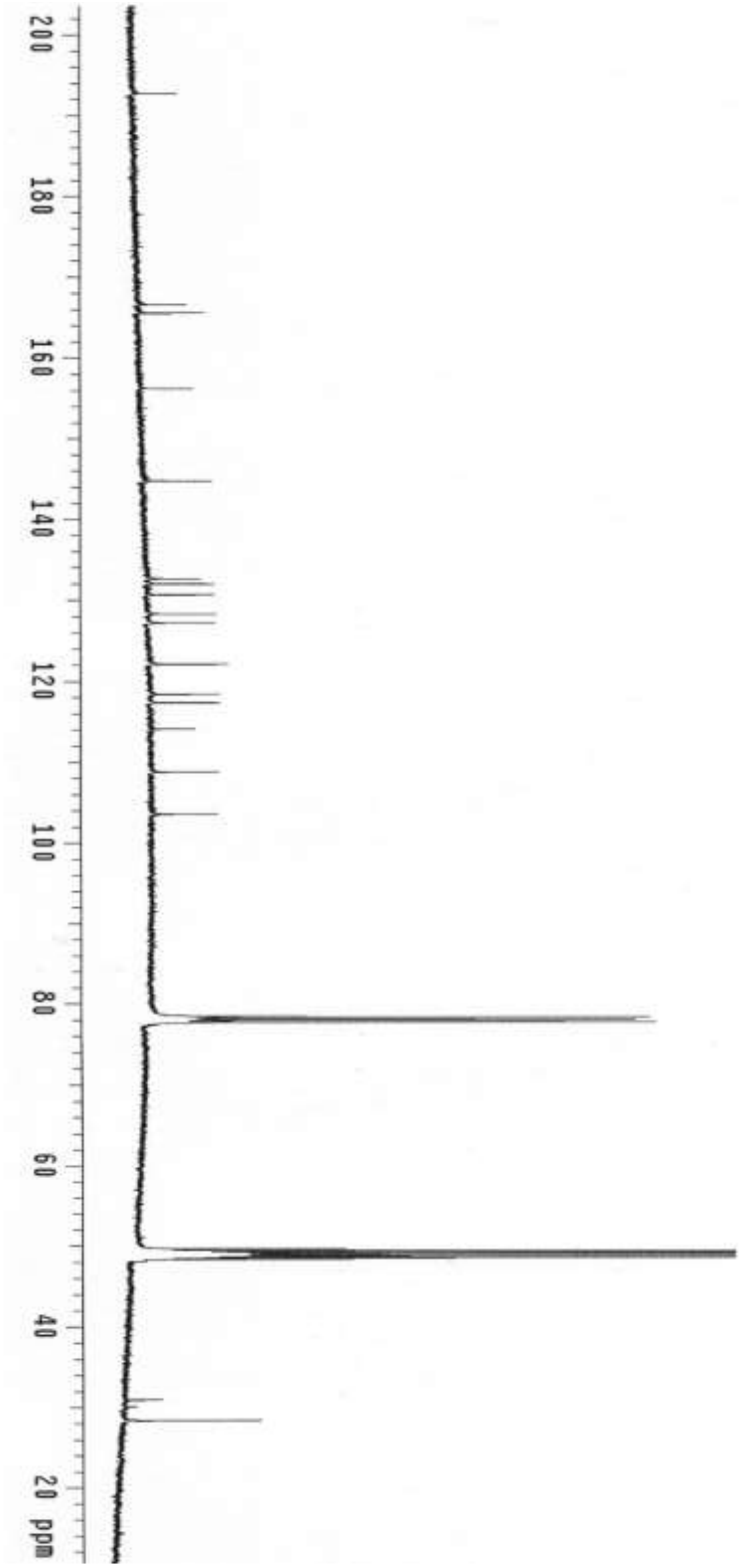

Figure S12. ^{13}C NMR spectrum of compound **3** (CD_3OD + $CDCl_3$, 100MHz)

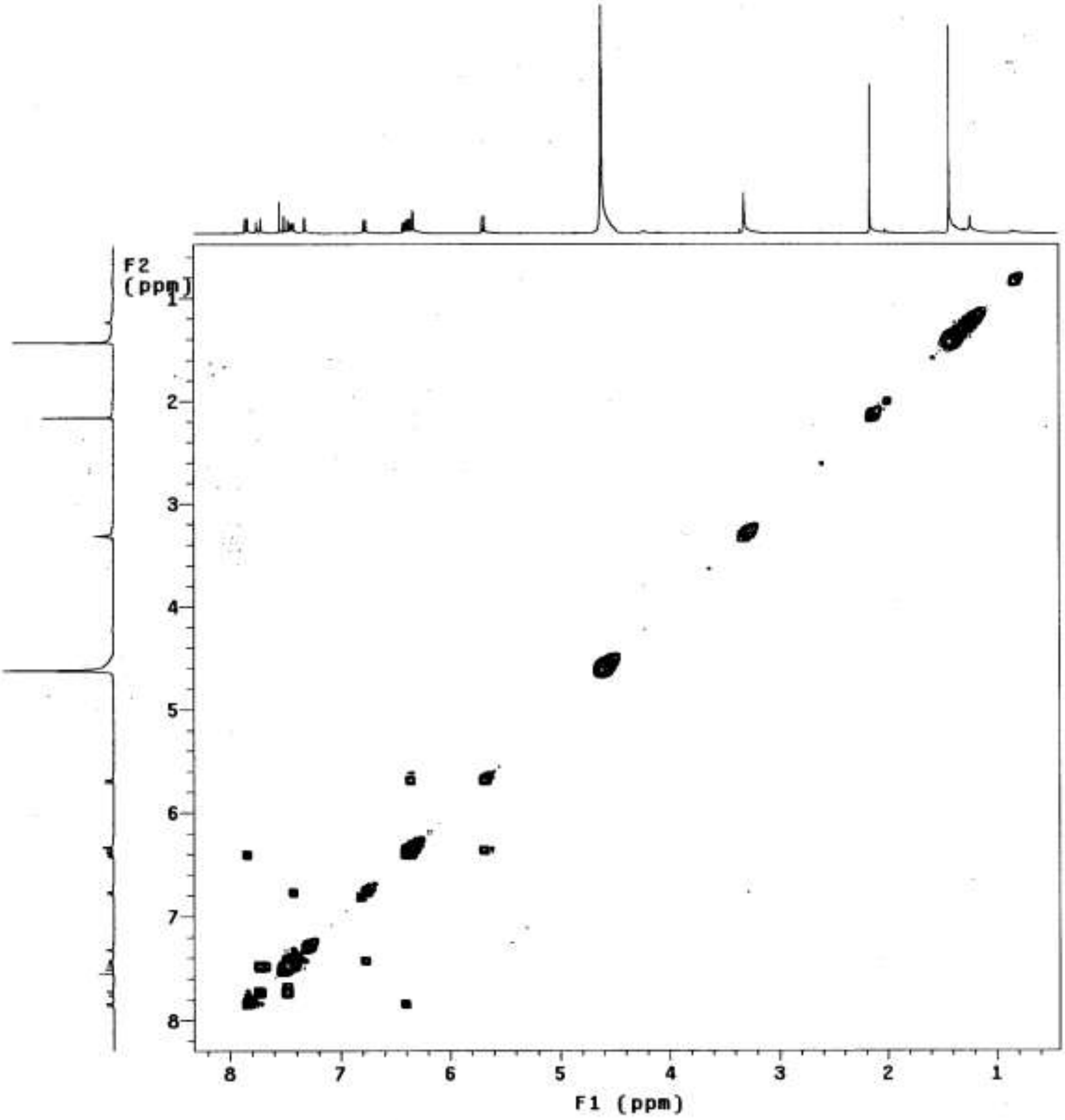

Figure S13. ^{1}H-^{1}H COSY spectrum of compound **3** (CD_3OD + $CDCl_3$, 400MHz)

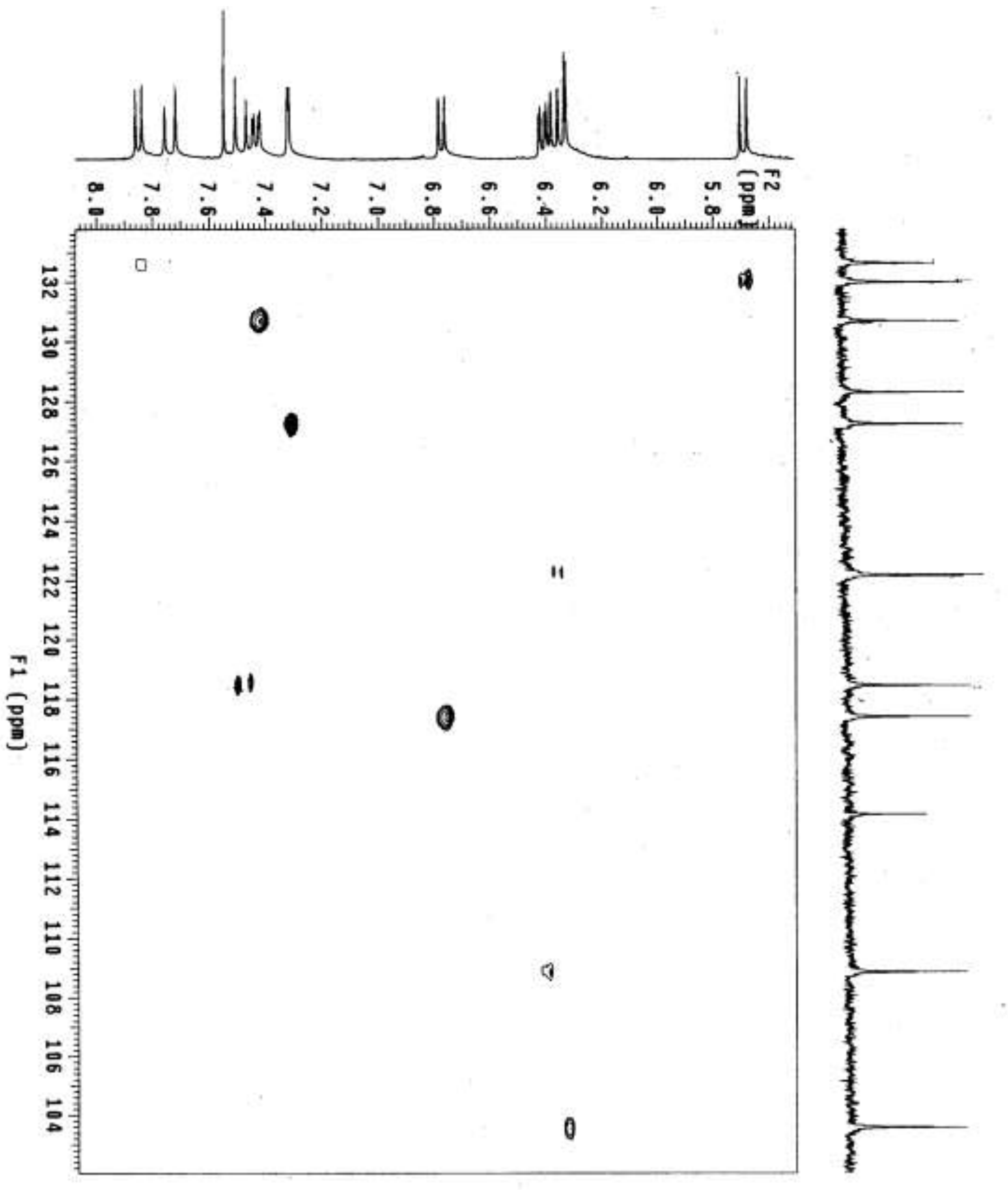

Figure S14. HSQC spectrum of compound **3** (CD_3OD + $CDCl_3$, 400MHz)

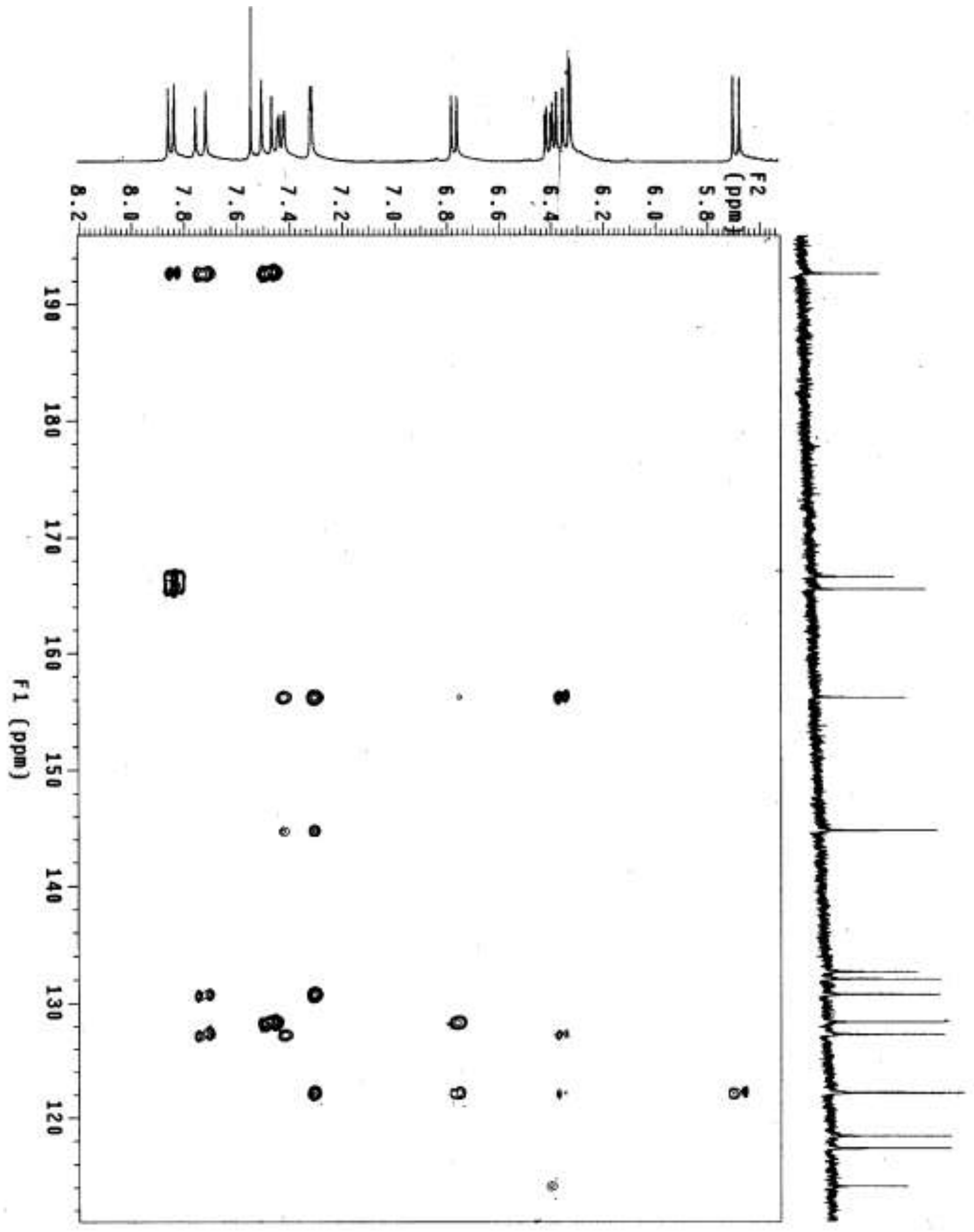

Figure S15. HMBC spectrum of compound **3** (CD_3OD + $CDCl_3$, 400MHz)
