# Supplementary material for: Modulation of Inducible Nitric Oxide Synthase Expression in LPS-Stimulated BV-2 Microglia by Prenylated Chalcones from Cullen corylifolium (L.) Medik. through Inhibition of I-κBα Degradation
Source: Molecules. 2018 Jan 4;23(1):109. doi: 10.3390/molecules23010109 (PMC6017879; doi:10.3390/molecules23010109)
Supplement: Supplementary file 1 [file molecules-23-00109-s001.zip › Supplementary materials Figure captions-proof reading-Ryu.docx]

**Supplementary materials**

**Figure captions**

**Figure S1.** ^1^H NMR spectrum of compound **1** (CD_3_OD, 400MHz)

**Figure S2.** ^13^C NMR spectrum of compound **1** (CD_3_OD, 100MHz)

**Figure S3.** ^1^H-^1^H COSY spectrum of compound **1** (CD_3_OD, 400MHz)

**Figure S4.** HSQC spectrum of compound **1** (CD_3_OD, 400MHz)

**Figure S5.** HMBC spectrum of compound **1** (CD_3_OD, 400MHz)

**Figure S6.** ^1^H NMR spectrum of compound **2** (CD_3_OD + CDCl_3_, 400MHz)

**Figure S7.** ^13^C NMR spectrum of compound **2** (CD_3_OD + CDCl_3_, 100MHz)

**Figure S8.** ^1^H-^1^H COSY spectrum of compound **2** (CD_3_OD + CDCl_3_, 400MHz)

**Figure S9.** HSQC spectrum of compound **2** (CD_3_OD + CDCl_3_, 400MHz)

**Figure S10.** HMBC spectrum of compound **2** (CD_3_OD + CDCl_3_, 400MHz)

**Figure S11.** ^1^H NMR spectrum of compound **3** (CD_3_OD + CDCl_3_, 400MHz)

**Figure S12.** ^13^C NMR spectrum of compound **3** (CD_3_OD + CDCl_3_, 100MHz)

**Figure S13.** ^1^H-^1^H COSY spectrum of compound **3** (CD_3_OD + CDCl_3_, 400MHz)

**Figure S14.** HSQC spectrum of compound **3** (CD_3_OD + CDCl_3_, 400MHz)

**Figure S15.** HMBC spectrum of compound **3** (CD_3_OD + CDCl_3_, 400MHz)
