# Supplementary figures and images for: Modulation of Inducible Nitric Oxide Synthase Expression in LPS-Stimulated BV-2 Microglia by Prenylated Chalcones from Cullen corylifolium (L.) Medik. through Inhibition of I-κBα Degradation
Source: Molecules. 2018 Jan 4;23(1):109. doi: 10.3390/molecules23010109 (PMC6017879; doi:10.3390/molecules23010109)

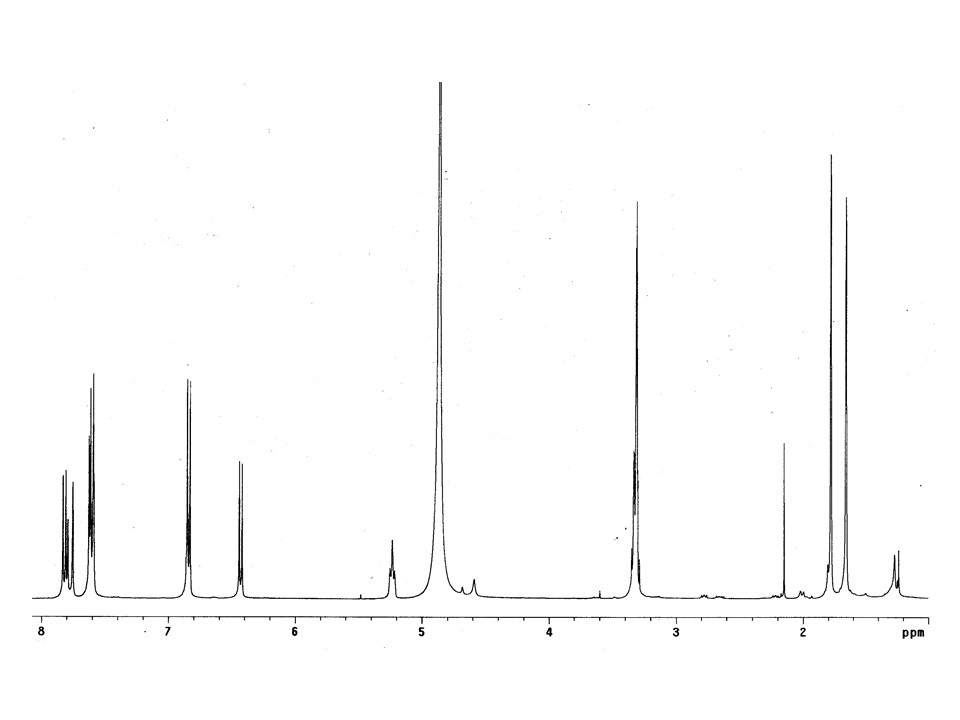

Supplement: Supplementary file 1 [file molecules-23-00109-s001.zip › Figure S1-1H NMR spectrum of compound 1.jpg]

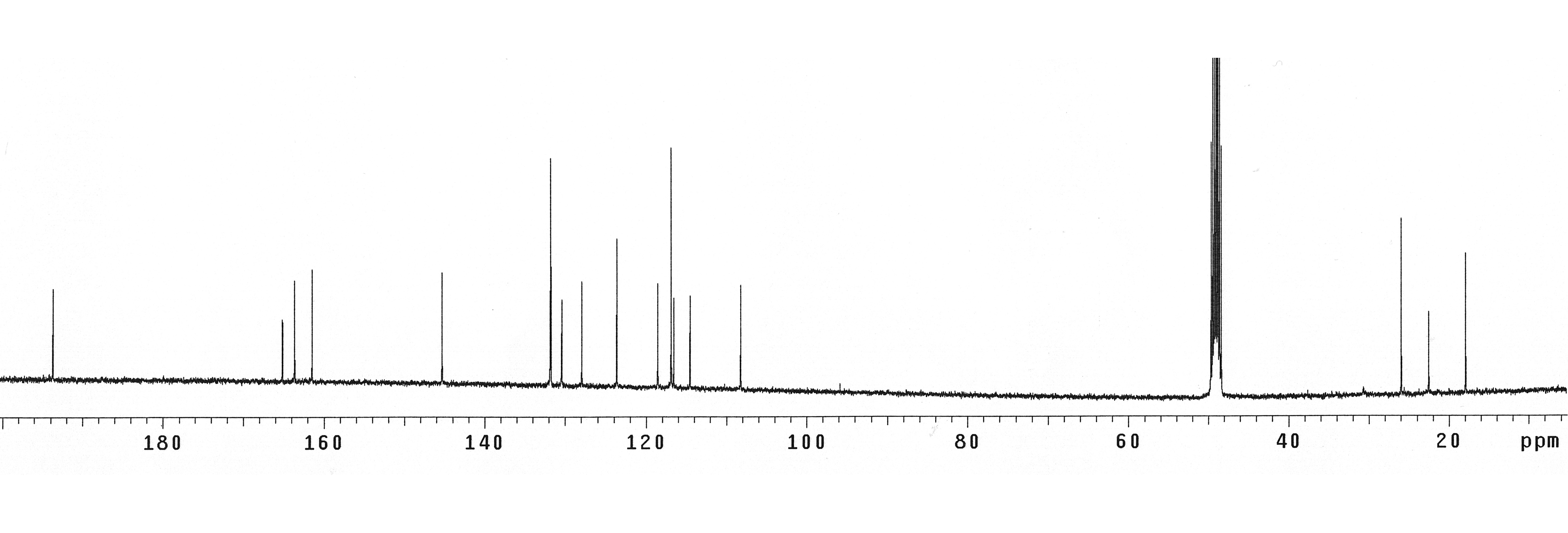

Supplement: Supplementary file 1 [file molecules-23-00109-s001.zip › Figure S2-13C NMR spectrum of compound 1.jpg]

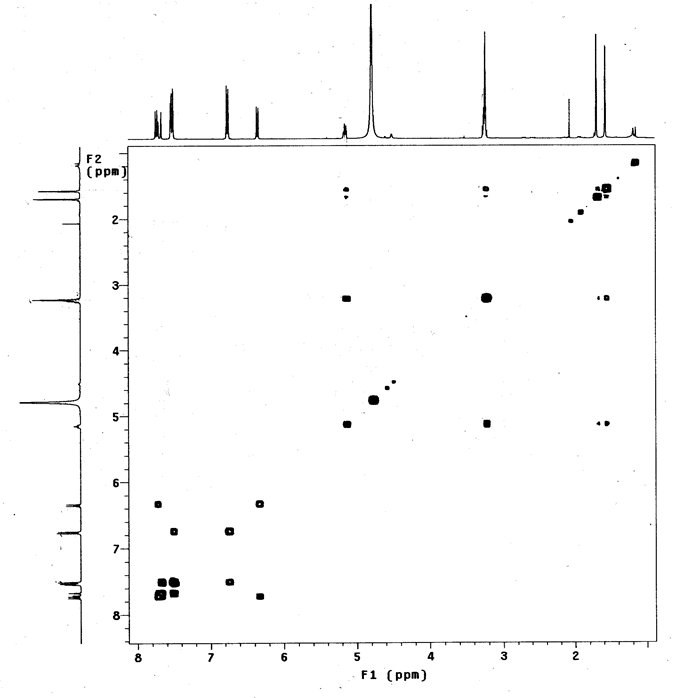

Supplement: Supplementary file 1 [file molecules-23-00109-s001.zip › Figure S3-1H-1H COSY spectrum of compound 1.jpg]

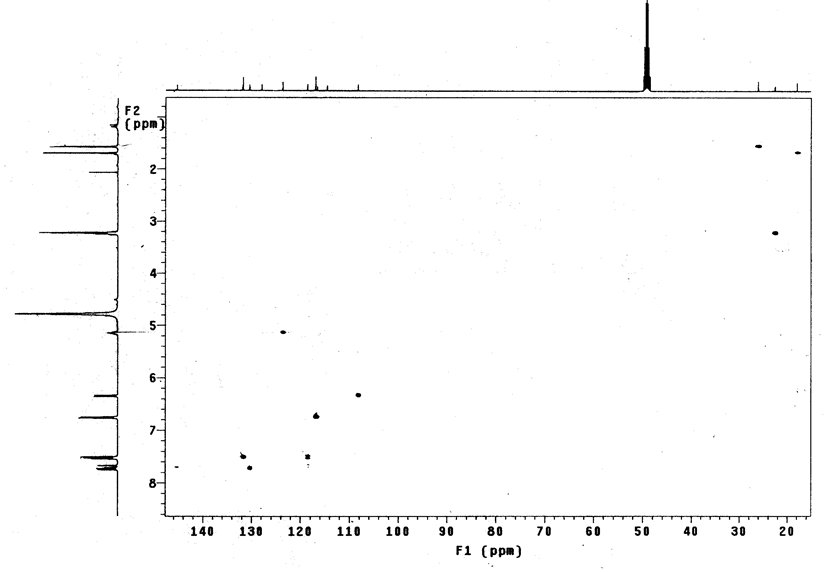

Supplement: Supplementary file 1 [file molecules-23-00109-s001.zip › Figure S4-HSQC spectrum of compound 1.jpg]

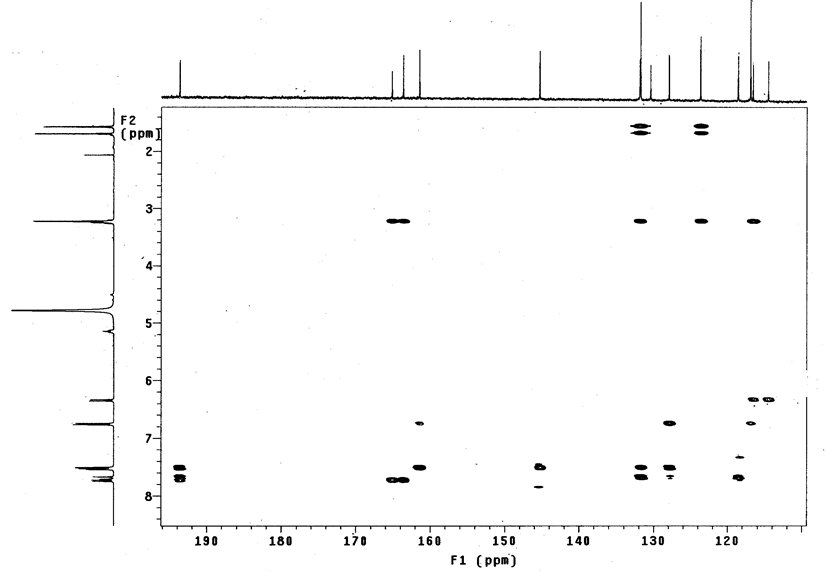

Supplement: Supplementary file 1 [file molecules-23-00109-s001.zip › Figure S5-HMBC spectrum of compound 1.jpg]

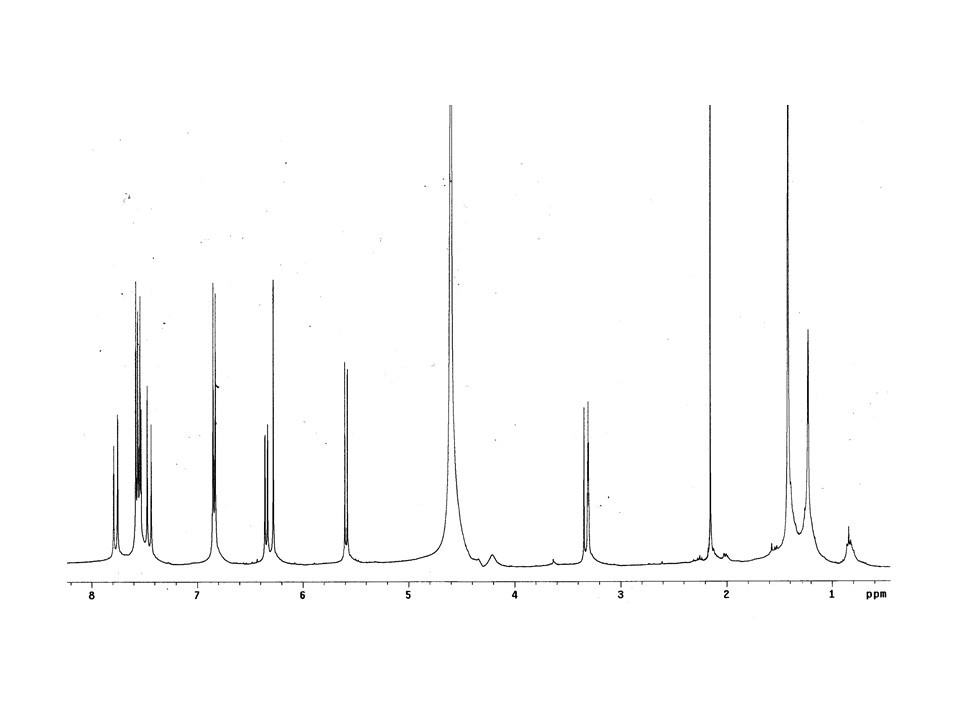

Supplement: Supplementary file 1 [file molecules-23-00109-s001.zip › Figure S6-1H NMR spectrum of compound 2.jpg]

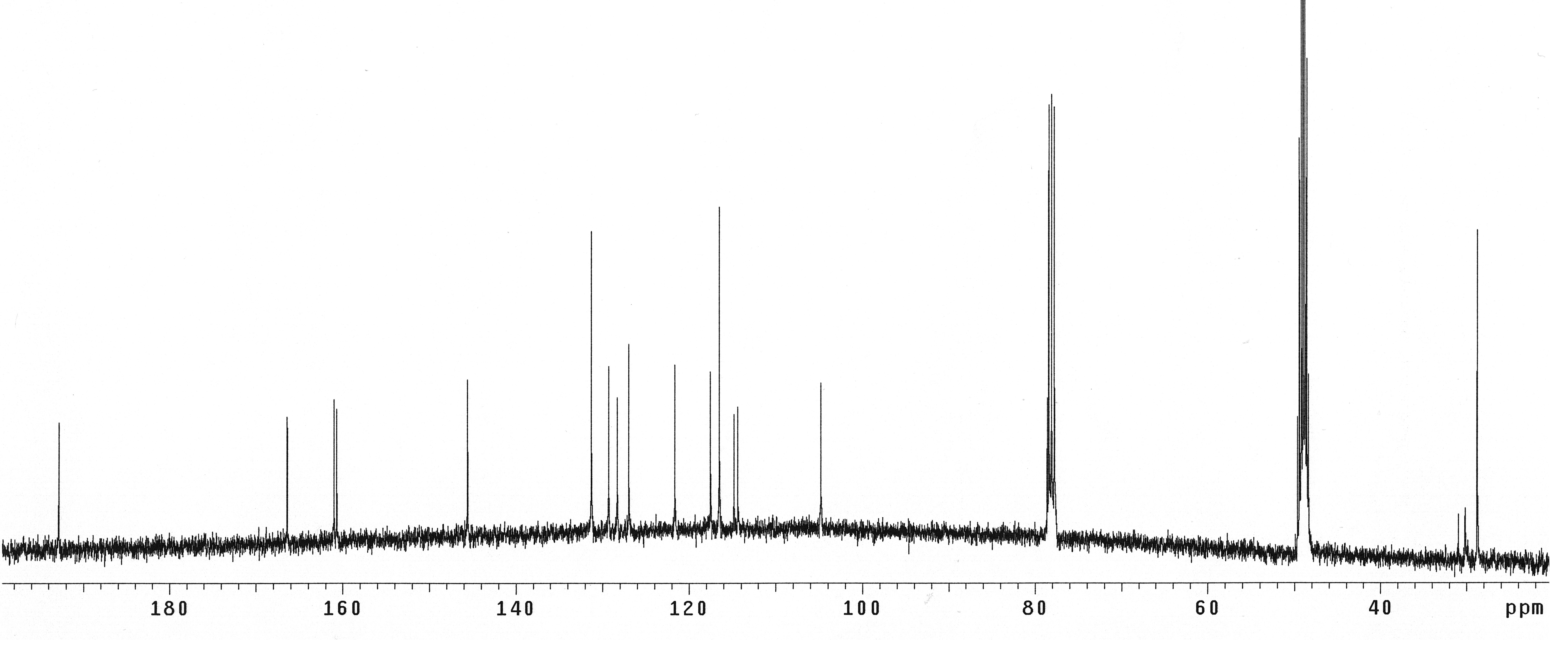

Supplement: Supplementary file 1 [file molecules-23-00109-s001.zip › Figure S7-13C NMR spectrum of compound 2.jpg]

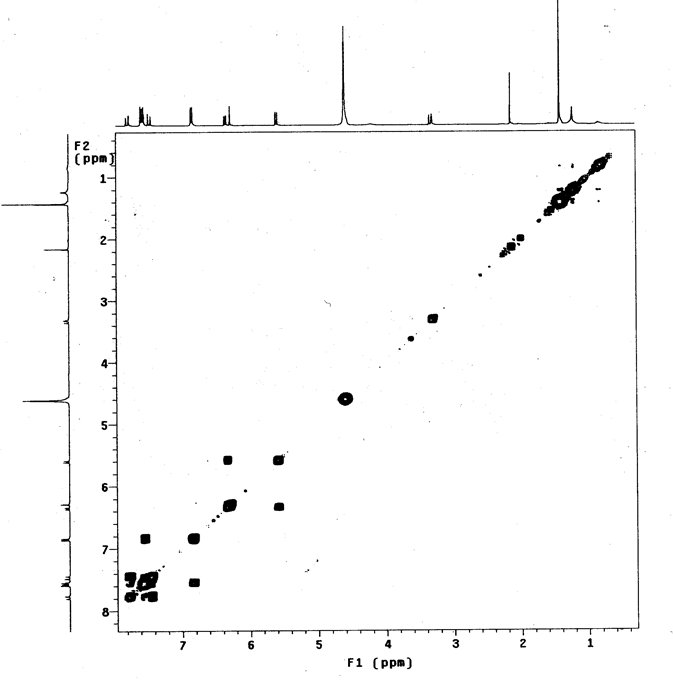

Supplement: Supplementary file 1 [file molecules-23-00109-s001.zip › Figure S8-1H-1H COSY spectrum of compound 2.jpg]

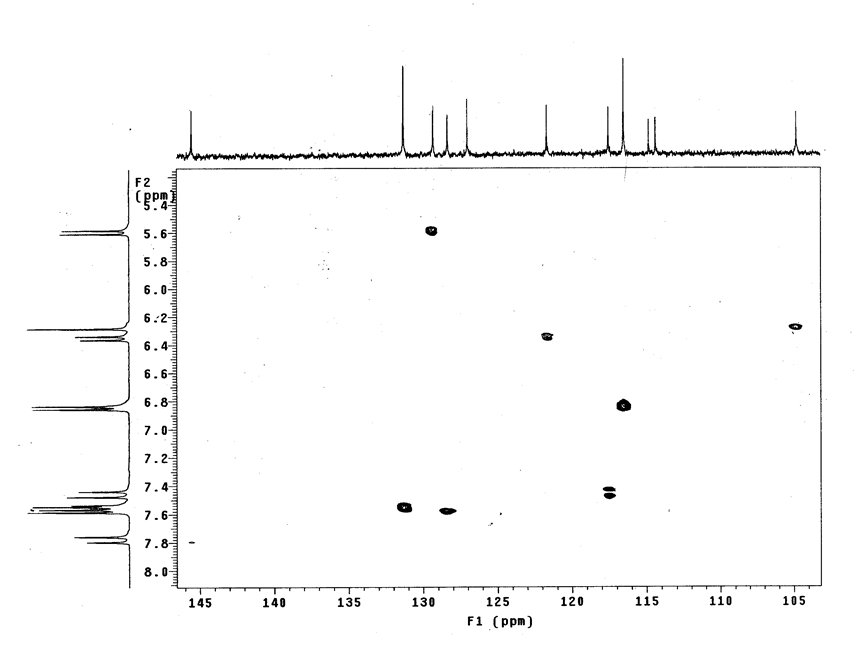

Supplement: Supplementary file 1 [file molecules-23-00109-s001.zip › Figure S9-HSQC spectrum of compound 2.jpg]

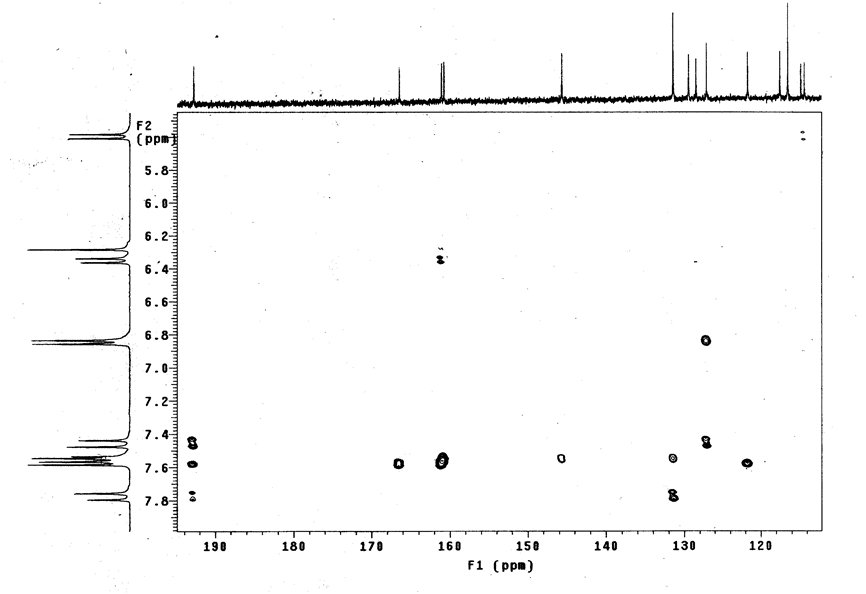

Supplement: Supplementary file 1 [file molecules-23-00109-s001.zip › Figure S10-HMBC spectrum of compound 2.jpg]

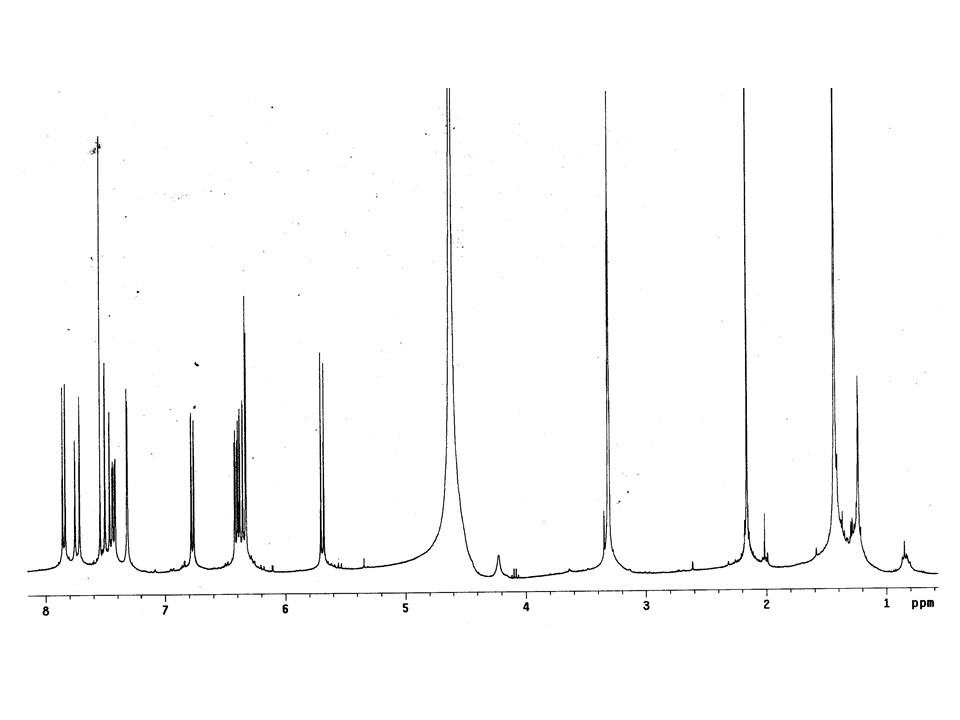

Supplement: Supplementary file 1 [file molecules-23-00109-s001.zip › Figure S11-1H NMR spectrum of compound 3.jpg]

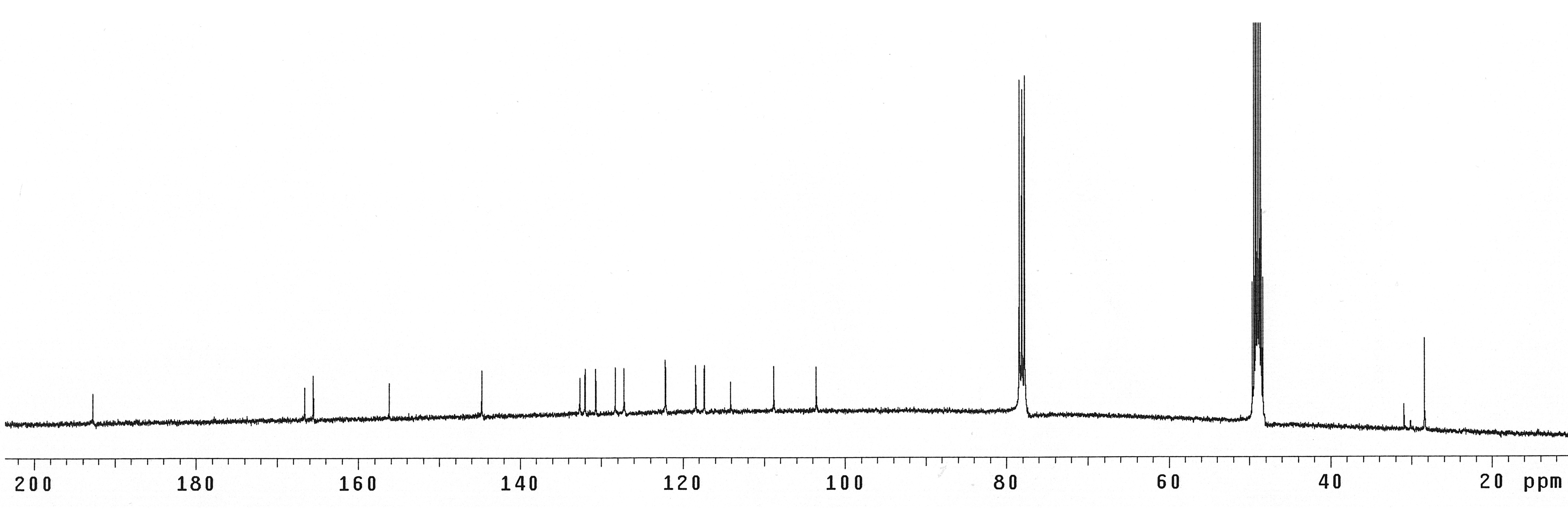

Supplement: Supplementary file 1 [file molecules-23-00109-s001.zip › Figure S12-13C NMR spectrum of compound 3.jpg]

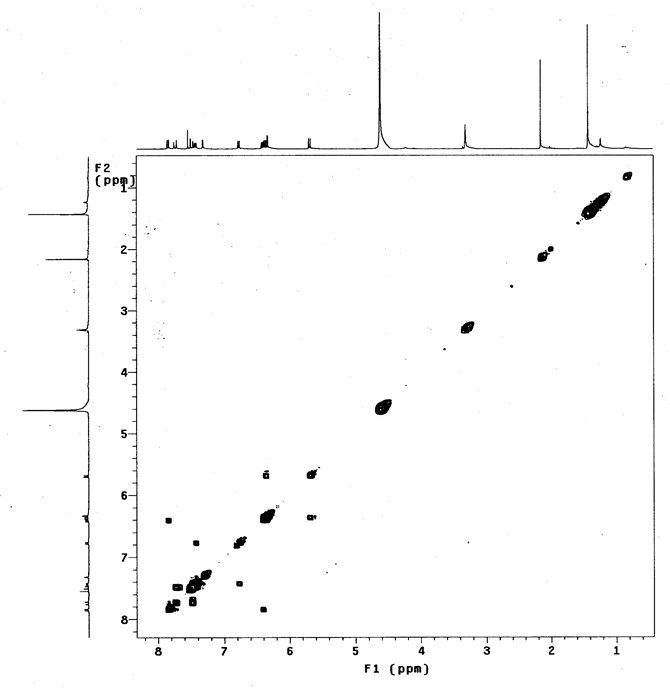

Supplement: Supplementary file 1 [file molecules-23-00109-s001.zip › Figure S13-1H-1H COSY spectrum of compound 3.jpg]

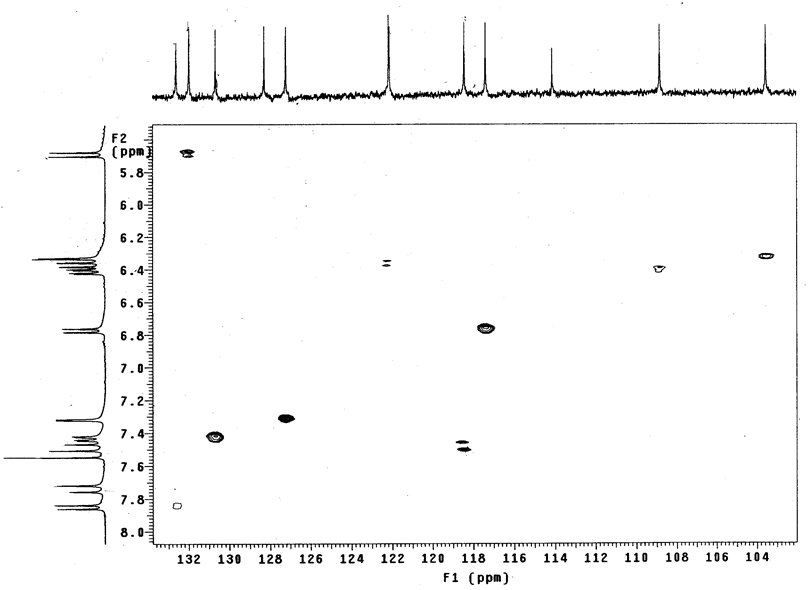

Supplement: Supplementary file 1 [file molecules-23-00109-s001.zip › Figure S14-HSQC spectrum of compound 3.jpg]

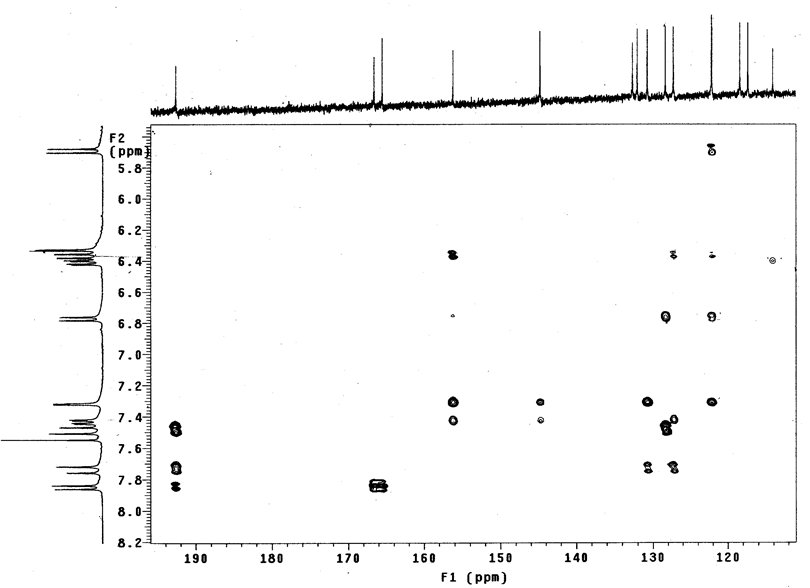

Supplement: Supplementary file 1 [file molecules-23-00109-s001.zip › Figure S15-HMBC spectrum of compound 3.jpg]
